# Proteomic analysis of low- and high-grade human colon adenocarcinoma tissues and tissue-derived primary cell lines reveals unique biological functions of tumours and new protein biomarker candidates

**DOI:** 10.1186/s12014-022-09364-y

**Published:** 2022-07-16

**Authors:** Matthew J. Munro, Susrutha K. Wickremesekera, Swee T. Tan, Lifeng Peng

**Affiliations:** 1 School of Biological Sciences and Centre for Biodiscovery, Victoria University of Wellington, Wellington, 6140 New Zealand; 2 Gillies McIndoe Research Institute, Newtown, PO Box 7184, Wellington, 6242 New Zealand; 3 Upper Gastrointestinal, Hepatobiliary & Pancreatic Section, Department of General Surgery, Wellington Regional Hospital, Wellington, 6021 New Zealand; 4 Wellington Regional Plastic, Maxillofacial & Burns Unit, Hutt Hospital, Lower Hutt, 5040 New Zealand; 5 Department of Surgery, The Royal Melbourne Hospital, The University of Melbourne, Melbourne, VIC 3050 Australia

**Keywords:** Biomarker, Colon adenocarcinoma, Colorectal cancer, Label-free quantitation, Proteomics

## Abstract

**Background:**

Colon cancer is the third most common cancer and second highest cause of cancer deaths worldwide. The aim of the study was to find new biomarkers for diagnosis, prognosis and therapeutic drug targets for this disease.

**Methods:**

Four low-grade and four high-grade human colon adenocarcinoma tumours with patient-matched normal colon tissues were analysed. Additionally, tissue-derived primary cell lines were established from each tumour tissue. The cell lines were validated using DNA sequencing to confirm that they are a suitable in vitro model for colon adenocarcinoma based on conserved gene mutations. Label-free quantitation proteomics was performed to compare the proteomes of colon adenocarcinoma samples to normal colon samples, and of colon adenocarcinoma tissues to tissue-derived cell lines to find significantly differentially abundant proteins. The functions enriched within the differentially expressed proteins were assessed using STRING. Proteomics data was validated by Western blotting.

**Results:**

A total of 4767 proteins were identified across all tissues, and 4711 across primary tissue-derived cell lines. Of these, 3302 proteins were detected in both the tissues and the cell lines. On average, primary cell lines shared about 70% of proteins with their parent tissue, and they retained mutations to key colon adenocarcinoma-related genes and did not diverge far genetically from their parent tissues. Colon adenocarcinoma tissues displayed upregulation of RNA processing, steroid biosynthesis and detoxification, and downregulation of cytoskeletal organisation and loss of normal muscle function. Tissue-derived cell lines exhibited increased interferon-gamma signalling and aberrant ferroptosis. Overall, 318 proteins were significantly up-regulated and 362 proteins significantly down-regulated by comparisons of high-grade with low-grade tumours and low-grade tumour with normal colon tissues from both sample types.

**Conclusions:**

The differences exhibited between tissues and cell lines highlight the additional information that can be obtained from patient-derived primary cell lines. DNA sequencing and proteomics confirmed that these cell lines can be considered suitable in vitro models of the parent tumours. Various potential biomarkers for colon adenocarcinoma initiation and progression and drug targets were identified and discussed, including seven novel markers: ACSL4, ANK2, AMER3, EXOSC1, EXOSC6, GCLM, and TFRC.

**Supplementary Information:**

The online version contains supplementary material available at 10.1186/s12014-022-09364-y.

## Background

Colorectal cancer (CRC) is the third most common cancer type, accounting for 10% of new cancer cases in 2020, and the second highest cause of cancer deaths [1]. Colon adenocarcinoma (CA) accounts for 90% of CRC cases [2]. CA grading has generally used a 2-tier system with low-grade CA (LGCA) comprising both well and moderately differentiated tumours, and poorly differentiated tumours regarded as high-grade CA (HGCA) [2].

Mutation of the *APC* gene is considered the initiating event of CRC development [3, 4]. Activating mutations of *KRAS* and deactivating mutations of *TP53* and *SMAD4* are also common in CRC, and their sequential accumulation contributes to the adenoma-carcinoma sequence of disease progression [5]. Metastasis is primarily driven by local invasion and subsequent entry of tumour cells into blood vessels located in or near the tumour, which then move through the circulation until they become trapped or adhere to capillaries in distant organs [6]. This movement is enabled by reduced cell adhesion and increased proteolysis [7].

CRC is typically diagnosed late due to it being asymptomatic at earlier stages of disease progression. Diagnosis is typically made by colonoscopy to visualise polyps or tumours, or faecal occult blood tests to detect blood in the stool [8]. Serum levels of carcinoembryonic antigen (CEA) are used as a prognostic tool for CRC patients and to indicate recurrence after treatment with 80% sensitivity and 70% specificity [9], although it can also be elevated in bowel inflammation, liver disease and pancreatitis [10]. An alternative blood test based on cell-free DNA, which is thought to be more abundant in cancer patients than healthy individuals, is in development [11]. Further pathologic testing is carried out by interrogating *KRAS*, *NRAS* and *BRAF* mutations and testing for mismatch repair (MMR) defects or microsatellite instability (MSI) [12]. MSI status has prognostic value, with MSI-H patients having better outcomes than microsatellite stable patients.

There is a need for more reliable, non-invasive and specific biomarkers to enhance detection of early-stage CRC, and treatments that target the mechanisms enabling CRC progression. Recently, there has been a focus on employing proteomics to identify CRC biomarkers. Interrogation of liquid biopsies from patients [13–15] identified fibronectin (FN1) and S100A9 as potential markers of CRC progression, and proposed that the combination of S100A9 and tenascin-C with CEA is superior to CEA alone as a CRC biomarker. A range of secretome and exosome biomarkers have been proposed, including C3a, APOC1, SERPINA1 and TSPAN1 [15–17]. Proteomics has previously been used to search for markers of cancer initiation or progression [18–21], and studies combining proteomics with genomics or transcriptomics have been performed on colon cancer tissue samples [22–27]. Commercial cell lines are often utilised for proteomic analyses, but use of primary tumour-derived cell lines is less common.

In this study, we analysed tumour tissue samples from 4 LGCA patients and 4 HGCA patients and their corresponding patient-matched normal colon (NC) tissue samples, and primary cell lines derived from the 8 CA tissue samples. Label-free quantitation (LFQ) proteomics was utilised to characterise the proteomes of the samples. The aims of this study were to characterise cell lines derived from colon tumour tissue in comparison with their tissues of origin, and to identify novel potential biomarkers and drug targets of CRC at tumour initiation and progression. The primary cell lines were validated using DNA sequencing and proteomics by comparison to their parental tissues. Differentially expressed proteins, measured by liquid chromatography tandem mass spectrometry (LC–MS/MS), were determined based on the confidence of protein identification and statistically significant changes in abundance between the patient-matched adjacent NC and LGCA and between LGCA and HGCA. The functional enrichments associated with these changes in the gene ontology (GO) terms, Kyoto Encyclopedia of Genes and Genomes (KEGG) and Reactome pathways were analysed to evaluate the samples at the system level. The proteomes and functional implications were also compared between CA tissues and CA-derived primary cell lines.

## Materials and methods

### Human colon tissue samples and tissue-derived primary cell lines

Formalin-fixed paraffin-embedded (FFPE) and snap-frozen tissue samples from 4 LGCA patients and 4 HGCA patients, with 8 patient-matched adjacent NC samples (4 from LGCA patients and 4 from HGCA patients), were provided by the Gillies McIndoe Research Institute Tissue Bank (GMRITB) for this study, which was approved by the Central Health and Disability Ethics Committee, Wellington, NZ (Ref. 15/CEN/96) with written informed consent from all patients. The FFPE tissue samples were used for immunohistochemical (IHC) staining. The snap-frozen tissues were used for proteomics, with a total of 16 tissue samples analysed. Patient clinical data can be found in Additional File 1.

Primary cell lines were derived from each of the CA tissues, giving 8 cell lines (4 from LGCA and 4 from HGCA) from different donors. Cell lines were provided by GMRITB at passage number 4–5 and were subcultured to a passage number not higher than 9 for experiments. Cells were cultured in Nunc™ EasYFlasks™ (ThermoFisher Scientific) using DMEM media (cat# 10569010, ThermoFisher Scientific) supplemented with 10% fetal calf serum (cat# 10091148, ThermoFisher Scientific), 5% mTeSR Complete (cat# 85850, STEMCELL Technologies, Tullamarine, Victoria, Australia), 1% penicillin–streptomycin (cat# 15140122, ThermoFisher Scientific) and 0.2% gentamicin/amphotericin B (cat# R01510, ThermoFisher Scientific). Cells were passaged upon reaching 75–95% confluency using TrypLE Express Enzyme (cat# 12605093, ThermoFisher Scientific).

### Immunohistochemistry

IHC staining was performed as previously described [28] on 4 µm sections of FFPE tissue samples with 3,3’-diaminobenzidine as the chromagen, using a Leica BOND Rx auto-stainer (Leica, Nussloch, Germany) using pre-defined protocols.

### DNA sequencing

DNA was extracted from FFPE tissue samples and tissue-derived cells using the PureLink Genomic DNA Mini Kit (cat# 1820-00, Invitrogen, ThermoFisher Scientific). The TaqMan™ RNase P Detection Reagents Kit (cat# 4316831, ThermoFisher Scientific) was used, as per the manufacturer’s instructions, to determine whether DNA was of sufficient quality to be successfully and accurately sequenced. DNA sequencing was performed by ThermoFisher Scientific (Life Technologies, Melbourne, Victoria, Australia) using the Oncomine™ TML assay to assess the mutational signatures of key CA-related genes in the CA tissues and the CA tissue-derived primary cell lines. DNA sequencing data were analysed using the cloud-based Ion Reporter system. Single-nucleotide variants and their impacts and tumour mutational burden were compared between FFPE tissues and the CA tissue-matched primary cell lines.

### Protein extraction

Tissues and cells were lysed in lysis buffer containing 30 mM Tris–HCl, pH 7.4; 7 M urea, 2 M thiourea, and 4% CHAPS plus 1% Halt™ Protease and Phosphatase inhibitor cocktail (cat# 78442, ThermoFisher Scientific), with a 1 mL glass Dounce homogeniser (Corning Inc, Corning, NY, USA) on ice. Samples were agitated for 45 min at 4 °C, then centrifuged at 17,000 g for 20 min at 4 °C. Protein concentration was measured by Bradford assay (Bio-Rad). The samples were stored at − 20 °C.

### Protein digestion and peptide purification

Extracted proteins were precipitated using a Calbiochem ProteoExtract® Protein Precipitation Kit as per manufacturer’s instructions (cat# 539180, Merck, Auckland, NZ). The protein pellet was dissolved in a digestion buffer of 8 M urea and 100 mM Tris–HCl, pH 8.5. After a second Bradford assay, 20 µg of protein was taken from each sample and the volume brought to 25 µL using digestion buffer. Subsequently, the proteins were reduced, alkylated and digested as previously described [29, 30]. The resulting tryptic peptides were purified using OMIX C18 100 µL zip-tips (cat# A57003100K, Agilent Technologies, Santa Clara, CA, USA). Eluates were pooled and dried down using a vacuum centrifuge, then brought to 100 µL using 0.1% formic acid (FA) in 3% acetonitrile (ACN).

### Liquid chromatography tandem mass spectrometry

Proteomic analysis of the prepared samples was performed by LC–MS/MS using an UlitMate 3000 RSLCnano system and a high-resolution Orbitrap Fusion™ Lumos™ Tribrid™ mass spectrometer coupled via a Nanospray Flex ion source (ThermoFisher Scientific). All samples were run individually.

Sample vials were placed in the autosampler of the HPLC unit for injection, maintained at 10 °C. Xcalibur™ software (Version 2.1.0, ThermoFisher Scientific) was used to define the method and acquire LC–MS/MS data. Peptides were first loaded onto an Acclaim™ PepMap™ 100 C18, 5 μm 0.3 × 5 mm trap column (cat# 160454, ThermoFisher Scientific) with 2% ACN and 0.05% trifluoroacetic acid at a loading pump flow rate of 8.0 µL/min, then separated on an Acclaim™ PepMap™ 100 C18, 2 µm, 100 A, 75 µm × 15 cm analytical column (cat# 164941, ThermoFisher Scientific) with the Nano/Cap pump running at 0.3 µL/min with an organic solvent gradient constructed from buffer A (0.1% FA) and buffer B (0.1% FA in 80% ACN). The gradient was programmed as follows: 3% from 0 to 5 min, 30% from 5 to 70 min, 50% from 70 to 82 min, 95% from 82 to 88 min, and finally 3% from 88 to 99 min. Elution was based on reverse-phase liquid chromatography, whereby the more hydrophobic the peptide is the slower it will pass through the column. Also, larger peptides have longer retention times.

The peptides eluted in solution were ionised by nanoelectrospray ionisation (Nanospray Flex, ThermoFisher Scientific) with the 25 µM Ion Transfer capillary tube set to 275 °C and voltage set at 1.8 kV. MS scans were acquired in the Orbitrap (OT) with the following settings: detector type OT, resolution 120,000, scan range 375–1500 m/z, AGC target 5.0e3, Maximum Injection Time 50 ms, charge state 2–7, and data type Profile. Data-dependent MS/MS scans were acquired in Ion trap having the following settings: detector type IT, scan range mode Auto: m/z Normal, IT scan rate Rapid, AGC target 5.0e3, Maximum Injection Time 300 ms, and data type Centroid. For MS/MS, high-energy collision-induced dissociation (HCD) fragmentation was performed in the linear Quadrupole ion trap (isolation window 1.6 m/z, HCD collision energy 30%). The “Top 20” highest-intensity ions from each MS scan were selected for the subsequent MS/MS scans. Dynamic exclusion had the following settings: mass tolerance 10 ppm, exclusion duration 60 s. Each sample was run in triplicate for LC–MS/MS.

### Protein identification

Spectra were exported as .raw files and searched against SwissProt human protein sequence database (TaxID = 9606 and Subtaxonomies, version 2017–10-25, downloaded on 24-10-2019, 42,253 sequences) using the SequestHT search engine in Proteome Discoverer (PD; version 2.4, ThermoFisher Scientific). Protein identification settings were as follows: peptide length range 6–144, allowing 2 missed trypsin cleavages, precursor mass tolerance 10 ppm, fragment mass tolerance 0.5 Da; cysteine carboxyamidomethylation static modification (+ 57.021); dynamic modifications included: oxidation (+ 15.995 Da) at M, carbamylation (+ 43.066 Da) at K, acetylation (+ 42.011 Da) at K, deamidation (+ 0.984 Da) at N and Q, peptide N-terminus modification of carbamylation (+ 43.066 Da). The files were searched against the protein sequence database and the decoy database (Percolator node) with a false discovery rate (FDR) of 0.01.

### Label-free quantitation

LFQ was carried out using PD, which has been demonstrated to perform better in terms of quantifiable low abundance proteome coverage than other search engines [31]. The .raw files for each individual sample in triplicate were imported, and each file was assigned to one category from each of 3 study factors: a “grade”, either NC or LG or HG; a “patient” number corresponding to biological replicates for each grade (from 1 to 4 for CA tissues and CA-derived cell lines, and from 1 to 8 for NC tissues); and a “technical replicate” number for each sample, numbered 1 to 3. Before running the analysis, comparisons were defined as LGCA / NC, HGCA / NC and HGCA / LGCA. At this stage, two analyses were run. For the first, all biological and technical replicates .raw files for each grade (NC, LGCA and HGCA) were pooled for quantitation analysis. For the second, the relative abundance of proteins in each individual CA tissue was compared with its patient-matched NC tissue.

Proteins with a minimum of one unique high-confidence peptide assigned were considered as positive identifications. Protein quantitation grouped abundances were used to calculate the abundance ratio for any given comparison. A t-test was performed by built-in statistical tools in PD to determine the statistical significance for each comparison. The selection criteria for significantly differentially expressed proteins were set at a fold-change (FC) of 2 or greater (log_2_ FC ≥ 1), and a p-value of 0.05 or smaller (-log_10_ p-value ≥ 1.30103). These significantly differentially expressed proteins were subsequently analysed for functional enrichments using STRING database (string-db.org) [32].

### Functional enrichment analysis

Significantly up-regulated or down-regulated proteins determined by LFQ were imported into STRING (string-db.org) [32] to identify protein–protein interaction networks and GO terms (“biological process”, “molecular function” and “cellular component”) and KEGG and Reactome pathways enriched in these networks. All STRING network analyses were performed with a medium confidence level (0.4). Nodes with no connections to other nodes under these criteria were removed from the map.

### Western blotting

Protein extraction and quantitation were performed as described above, except lysis buffer was Pierce™ RIPA buffer (cat# 89901, ThermoFisher Scientific, Waltham, MA, USA).

Protein samples were diluted in 1 × Bolt LDS sample buffer (cat# B0007, ThermoFisher Scientific), heated at 85 °C for 5 min and run on Bolt 4–12% Bis–Tris gels (cat# NW04125BOX, ThermoFisher Scientific) with 20 µg of sample protein per lane, and 1–2 µL of Precision Plus Protein Kaleidoscope MW ladder (cat# 1610375, Biorad, Auckland, NZ) run in the first lane. Gels were run in Bolt MES SDS Running Buffer (cat# B0002, ThermoFisher Scientific) for 50 min at 150 V, 3.00A and 300 W.

Electrophoresed proteins were transferred to PVDF membranes using an iBlot 2 apparatus (cat# IB21001, ThermoFisher Scientific), briefly washed in water and blocked using iBind Flex FD solution (cat# SLF2019, ThermoFisher Scientific) before exposure to primary and secondary antibodies in an iBind apparatus (cat# SLF1000 or SLF2000, ThermoFisher Scientific). Primary antibodies included: α-tubulin (1:2000; cat# ab7291, Abcam), CD44 (1:5000; cat# ab157107, Abcam), FN1 (1:500; cat# ab2413, Abcam), and S100A9 (1:500; cat# ab92507, Abcam). Secondary antibodies included: HRP-linked goat anti-rabbit (1:1000; cat# ab6721, Abcam), HRP-linked goat anti-rabbit (1:1000; cat# 111-035-045, Jackson Immunology), and Alexa Fluor® 488 donkey anti-mouse (1:1000; cat# A-21202, ThermoFisher Scientific). Membranes were incubated in the iBind apparatus for 2.5 h or overnight. Following incubation, the membranes were briefly washed in water and developed using Clarity Western ECL Substrate (cat# 170-5061, Biorad). Imaging and densitometry were performed using a ChemiDoc MP Imaging System (Biorad) and ImageLab 6.0 software (Biorad), with the intensity values for the protein-of-interest normalised against α-tubulin and analysed using GraphPad Prism 8 (San Diego, CA, USA).

## Results

### Immunohistochemical staining of tissues

IHC staining demonstrated the branching crypts of LGCA tumours and the chaotic architecture of the HGCA tumours (Additional File 2).

### DNA sequencing of CA tissues and CA tissue-derived cell lines

DNA of sufficient quality, defined as having a 260/280 absorbance ratio of ~ 1.8 (NanoDrop 2000, ThermoFisher Scientific) and confirmed using the TaqMan™ RNase P Detection Reagents Kit (cat# 4316831, ThermoFisher Scientific) according to the manufacturer’s instructions, was isolated from 3 of the 4 LGCA and all 4 HGCA patient-derived tissue and cell line samples, and the DNA samples were analysed by DNA sequencing. The pre-defined Oncomine Tumor Mutation Load w3.2 analysis workflow was launched for each individual tissue and cell line sample, which calculated the tumour mutational burden (TMB) score for each sample, identified and annotated variants within 409 cancer-related genes, and assigned an impact level to each variant. Following this, paired analyses were performed to compare each tissue with its matched cell line.

To confirm whether the mutations detected in the cells were the same as those in the tissues and thereby determine the similarity of the cell lines to their parent tissues, several CA-related genes were selected and the specific mutations within these genes were explored (Table 1). The *APC*, *TP53*, *KRAS*, *BRAF*, *PIK3CA* and *FBXW7* genes were identified by other studies as being relevant to CRC [4, 23, 33]. *MSH2*, *MSH6* and *MLH1* genes are vital to DNA MMR and MSI, and mutations to these genes increase the risk of CRC development by approximately 80% [3, 4].

**Table 1:** Mutations to CA-related genes in FFPE CA tissues and CA-derived primary cell lines

Gene	Number of sites mutated	Total mutations	Shared mutations between tissue and matched primary cell line
*APC*	12	41	26
*TP53*	7	10	5
*KRAS*	2	7	4
*BRAF*	2	4	4
*MSH2*	2	3	3
*MSH6*	11	26	20
*MLH1*	3	8	7
*PIK3CA*	4	11	5
*FBXW7*	2	2	2

The *APC* gene was the most commonly mutated, with the c.5034 G > A mutation present in the tissues and cell lines for all 7 samples. A total of 41 *APC* mutations were identified across all samples at 12 different sites (Table 1). Of the 41 mutations, 32 were detected across all tissue samples and 34 across all cell lines, with 26 of these shared by a tissue sample and its matched cell line (Additional File 3). *TP53* and *MSH6* were also frequently mutated. Half of the total number of mutations detected in the *TP53* gene were shared between sample types, and half were only present in the tissues but not in the tissue-derived cell lines. However, there was only one site that was mutated in more than one patient, that being c.215 C > A which was detected in both the tissues and cell lines derived from 4 patients (Additional File 3). Similarly, *PIK3CA* mutations were shared in less than 50% of cases, due to mutations arising in the cell lines that were absent in the tissues. Overall, 76 out of 112 (67.9%) mutations were shared by the CA tissue samples and the matched CA-derived cell lines based on the analysis of these 9 genes (Table 1). While the CA tissue samples were variable in terms of their unique mutations and TMB scores (Additional File 4), the cell lines derived from them displayed a more consistent number of mutations. The analysis carried out here is similar to another recently performed in meningioma, where the authors expected large variations between cell lines and their parent tissues after 10 passages in culture [34]. Therefore, given that the CA-derived cell lines had been in culture for up to 9 passages, the overlap of 67% of detected mutations between the tissues and cell lines in key CA-related genes suggests that the cell lines are a suitable in vitro model of the tumour tissue.

DNA sequencing results from 9 key CRC-related genes were analysed in 7 CA tissues and their 7 patient-matched tissue-derived primary cell lines. The number of sites mutated and the total number of mutations across all samples at those sites are listed, as well as the number of mutations that were shared by a tissue sample and its matched cell line. Mutations with low frequency (< 6%) and/or coverage depth (< 300 reads), which were unique to 1 sample type (either the tissues or cell lines from a patient, but not both) and/or unique to 1 patient, were considered to be read errors and were disregarded.

### Protein identification

A total of 4767 high-confidence proteins were identified across all tissue samples (4 HGCA, 4 LGCA, 8 NC; 3 LC–MS/MS replicates per biological sample), and a total of 4711 high-confidence proteins were identified across cell lines (4 HGCA-derived, 4 LGCA-derived; 3 LC–MS/MS replicates). Of the proteins identified, 3302 proteins were shared by the pooled tissues and pooled cell lines, accounting for 69.3% and 70.1% of the proteins identified in the tissues and cell lines, respectively. This was also the case when comparing the proteomes of each primary cell line with its parent tumour tissue sample individually, with around 70% of proteins detected in one sample type also seen in the other. Complete lists of the proteins identified from tissue samples and from cell lines are available in Additional Files 5 and 6, and a list of all proteins shared by the tissues and cell lines can be found in Additional File 7. The raw LC–MS/MS data have been deposited to ProteomeXchange Consortium [35] via the PRIDE [36] partner repository with the dataset identifier PXD024449.

The similarities of biological replicates for each condition were assessed for the tissues and cell lines. The 4 LGCA-derived cell lines shared 56.9% of identified proteins (Additional File 8A), while the 4 HGCA-derived cell lines shared 34.4% (Additional File 8B). NC tissues from LGCA and HGCA patients shared 31.4% and 26.9% of identified proteins, respectively (Additional File 8C, D). The 4 LGCA and 4 HGCA tissues shared 50.7% and 51.0% of proteins, respectively (Additional File 8E, F). This reflected the molecular heterogeneity between patients.

One strength of this study is the proteomic analysis of tissue-derived primary cell lines compared to their parent tissues, a point of difference from most proteogenomic studies which tend to analyse tissue samples, commercial cell lines or serum samples. The protein identification data partially corroborated the DNA sequencing data by confirming that the LGCA-derived cell lines had a higher degree of similarity to each other than did the tissue samples, representing a purer population compared with the heterogeneous nature of the tissue. However, the HGCA-derived cell lines were the most variable cancer sample group.

### Differentially expressed proteins

LFQ identified proteins that displayed significantly differential expression in LGCA and HGCA tissues versus patient-matched NC tissues, HGCA tissues versus LGCA tissues, and HGCA-derived cell lines versus LGCA-derived cell lines (Table 2). Overall, 318 proteins were significantly upregulated, whereas 362 proteins were significantly downregulated, which represent the proteomic signatures of CA development from the initiation to an advanced stage. The details of these proteins and their quantitation data can be found in Additional Files 5 and 6. Proteins of interest that were selected based on their functions relevant to tumorigenesis, signalling and regulation, and immune responses are listed in Table 3.

**Table 2:** Number of proteins with significantly different relative abundances between each group (HGCA, LGCA and NC) for CA tissues and CA-derived cell lines

Comparison		Number of upregulated proteins	Number of downregulated proteins
Tissues	HGCA / LGCA	97	91
	LGCA / LGNC	88	121
	HGCA / HGNC	104	168
Tissue-derived cell lines	HGCA / LGCA	137	123

**Table 3:** Selected proteins with differential expression identified in the CA tissues and/or CA tissue-derived primary cell lines

Gene symbol	Protein name	Abundance ratio (p-value)				References for involvement in CA
		Tissues			Cell lines	
		LG/NC	HG/NC	HG/LG	HG/LG	
ACE2	Angiotensin-converting enzyme 2	**100 (< 0.001)**	**100 (< 0.001)**	0.725 (0.703)	–	62,63
ACSL4	Long-chain-fatty-acid-CoA ligase 4	1.682 (0.993)	2.403 (0.996)	1.533 (0.946)	**2.022 (0.01)**	–
ANK2	Ankyrin-2	**0.486** **(0.03)**	**0.219** **(< 0.001)**	0.454 (0.056)	–	–
AMER3	APC membrane recruitment protein 3	–	–	–	**0.485 (0.039)**	–
CD44	CD44 antigen	1.705 (0.973)	2.263 (0.991)	1.264 (0.999)	**2.269 (< 0.001)**	55
CIRH1A (UTP4)	U3 small nucleolar RNA-associated protein 4	**100** **(< 0.001)**	**100** **(< 0.001)**	1.693 (0.946)	-	68
CNN1	Calponin-1	**0.369 (0.046)**	**0.22** **(0.002)**	0.699 (0.305)	0.735 (0.457)	72–74
EXOSC1	Exosome complex component csl4	**100 (< 0.001)**	**100 (< 0.001)**	1.196 (0.999)	0.982 (0.983)	–
EXOSC6	Exosome complex component MTR3	**100 (< 0.001)**	**100 (< 0.001)**	1.189 (0.999)	1.34 (0.936)	–
FN1	Fibronectin	1.429 (0.963)	**3.32 (0.986)**	**2.358 (0.076)**	**0.35 (< 0.001)**	14,49,50
GCLM	Glutamate–cysteine ligase regulatory subunit	–	–	–	**2.723** **(0.004)**	–
HMOX1	Heme oxygenase-1	–	–	–	**3.447 (0.002)**	43
MSH6	DNA mismatch repair protein MSH6	**0.444 (0.024)**	**0.304** **(0.001)**	**1.837 (0.775)**	2.107 (0.422)	3,4
S100A8	Protein S100-A8	1.66 (0.956)	4.417 (0.897)	**2.434 (0.026)**	–	18,23
S100A9	Protein S100-A9	1.807 (0.984)	4.855 (0.878)	**2.524** **(0.02)**	–	14,18,23
SDCBP	Syntenin-1	1.565 (0.984)	4.929 (0.781)	**2.798** **(0.01)**	1.547 (0.549)	54
SFRP4	Secreted frizzled-related protein 4	2.031 (0.963)	8.834 (0.093)	**3.747 (0.008)**	–	52
SMC2	Structural maintenance of chromosomes protein 2	**100 (< 0.001)**	**100 (< 0.001)**	2.436 (0.169)	1.459 (0.831)	58,59
TFRC	Transferrin receptor protein-1	2.621 (0.956)	5.358 (0.785)	1.847 (0.391)	**2.02 (0.005**)	–

Values represent the abundance ratios, with p-value in brackets. Significantly differential abundance defined as FC > 2 and p < 0.05. Numbers in bold represent significant upregulation or downregulation. “–“ = not detected

All the NC samples are parent-matched with the LGCA or HGCA tumour samples from 8 patients (4 LGCA and 4 HGCA).

### Functional enrichment analysis of differentially expressed proteins in CA tissues

**Fig. 1:**
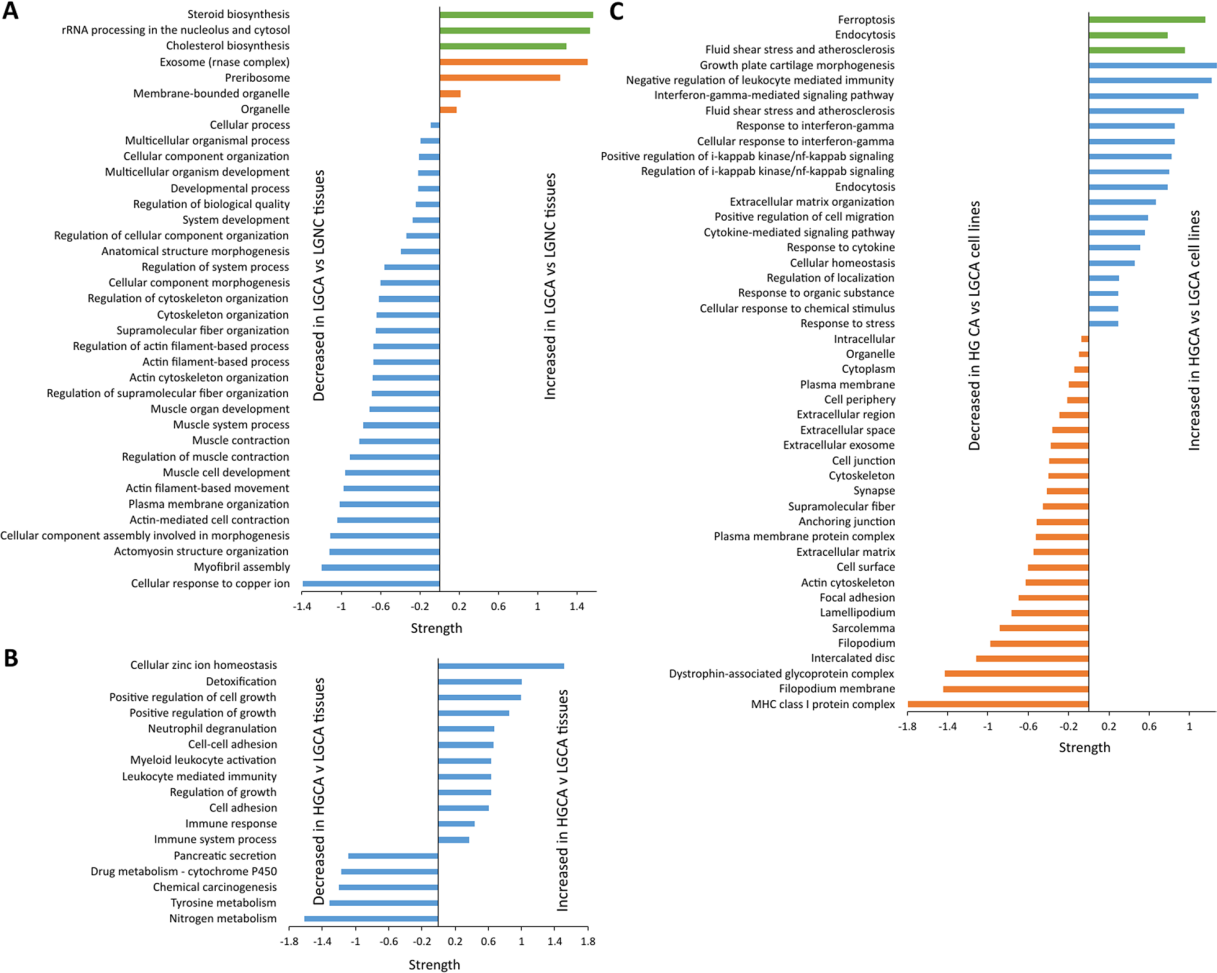
Functional enrichments of proteins with significantly differential abundance in CA. **A** LGCA v LGNC tissues. **B** HGCA v HGCA tissues. **C** HGCA v LGCA cell lines. The analyses were performed using STRING online software with a medium confidence level (0.4), providing the significantly enriched GO terms, KEGG and Reactome pathways. The vertical axis displays the significantly enriched biological functions. The horizontal axis indicates the strength of enrichment, which is the log10 (observed/expected proteins in the network that are annotated with a term) and measures how large the enrichment effect is. The higher the absolute value of the strength, the stronger the impact. Positive strength values indicate enrichment amongst significantly upregulated proteins, negative strength values indicate enrichment amongst significantly downregulated proteins. The lists of differentially expressed proteins used to create these graphs are found in Additional Files 5 and 6. Colour codes: the green denotes KEGG and Reactome Pathways; the orange and blue code GO Cellular Component and Biological Process categories, respectively

The GO terms and KEGG and Reactome pathways enriched in the initiation (NC to LGCA) and progression (LGCA to HGCA) of tumours are summarised in Fig. 1. Networks containing proteins or pathways of interest are displayed in Additional Files 9–11.

Relative to NC tissues, the proteins overexpressed in LGCA tissues were enriched for preribosome activity, components of the RNA-degrading exosome complex and intracellular organelles (Fig. 1A, Additional File 9A), indicating elevated RNA processing and translational capacity for biosynthesis. Indeed, the data showed that sterol and cholesterol lipid biosynthesis increased (Additional File 9A). Conversely, proteins with significantly decreased abundance in LGCA relative to NC tissues were mainly involved in cytoskeletal organization, cellular component morphogenesis, muscle system processes, and the caveolar macromolecular signalling complex (Fig. 1A, Additional File 9B).

The proteins with significant overexpression in HGCA tissues relative to LGCA tissues were enriched for immune system responses, detoxification and cell adhesion (Fig. 1B, Additional File 10A). Among this network, FN1 was a key node. Unsurprisingly, enriched GO terms included the positive regulation of cell growth (Fig. 1B). HGCA tissues displayed significant decreases relative to LGCA in proteins involved in metabolism of nitrogen, tyrosine and drugs via cytochrome P450 (Fig. 1B, Additional File 10B).

### Functional analysis of differentially expressed proteins in CA tissue-derived primary cell lines

Proteins with significantly increased abundance in HGCA cells relative to LGCA cells were enriched for IFN-g and NF-kB signalling, cytokine-mediated signalling, ferroptosis and endocytosis (Fig. 1C, Additional File 11A). Key ferroptosis markers present in this network included long-chain-fatty-acid-CoA ligase 4 (ACSL4), transferrin receptor 1 (TFRC), heme oxygenase 1 (HMOX1), and glutamate-cysteine ligase modifier (GCLM). Proteins upregulated in HGCA cell lines that contribute to IFN-g signalling included guanylate-binding protein 1 (GBP1), vascular cell adhesion protein 1 (VCAM1), MHC class I components (HLA-A69, HLA-B), and the proposed stem cell marker CD44. HGCA cell lines displayed significant reductions of membrane-associated proteins including other MHC class I components (HLA-A24, HLA-A30, HLA-B41), dystrophin-associated glycoprotein complexes (PGM5, SGCD, SNTB1), filopodia-associated proteins (HLA-G, ITGA3, GAP43), and proteins involved in cell adhesion, cell junctions and cytoskeleton (Fig. 1C, Additional File 11B).

### Western blotting

**Fig. 2:**
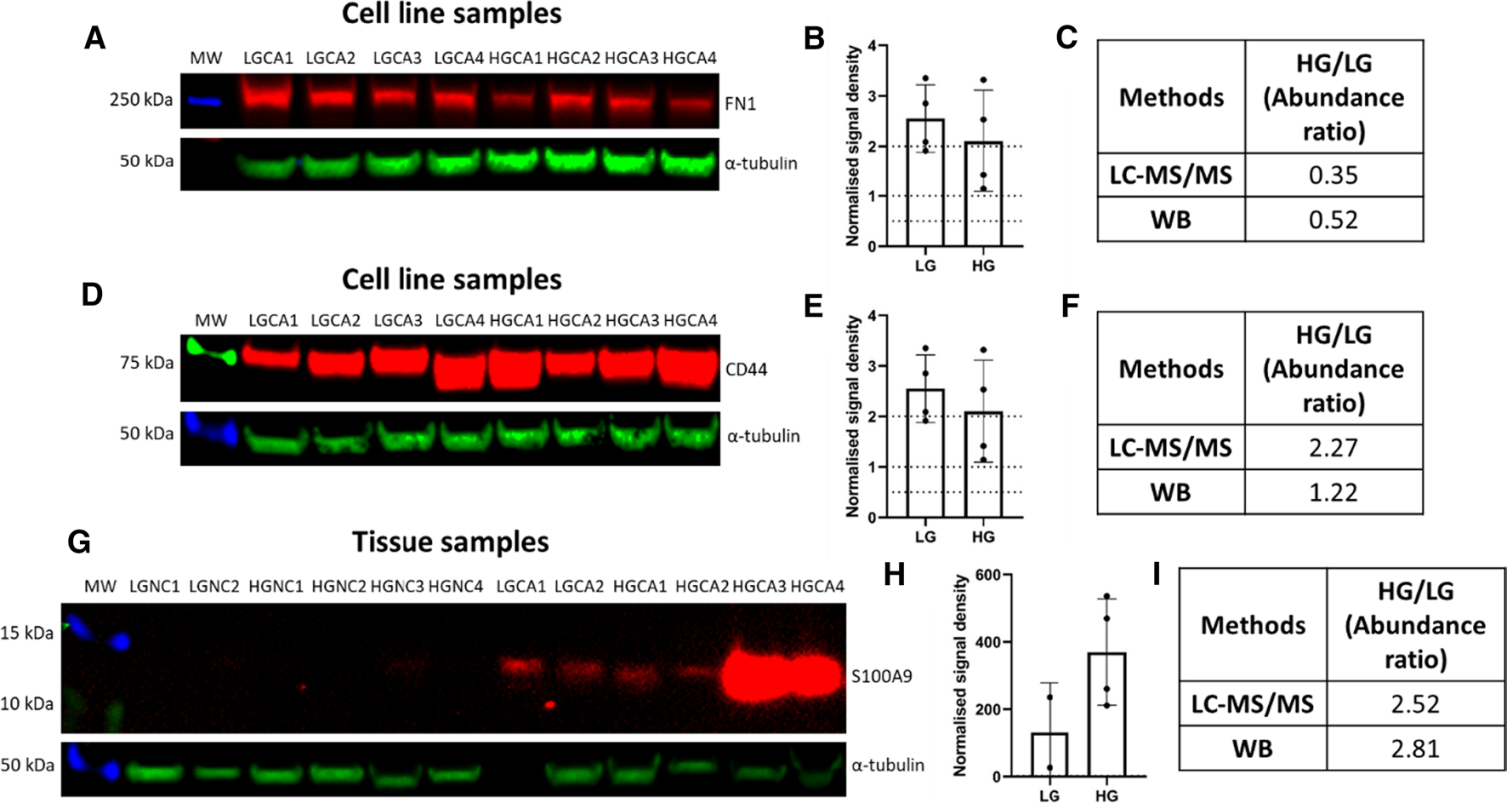
Western blotting for FN1 and CD44 proteins extracted from CA-derived cell line samples and S100A9 from CA tissue samples. Fluorescent signals for FN1 (**A**; red), CD44 (**D**; red) and S100A9 (**G**; red), and the loading control α-tubulin (**A**, **D**, **G**; green). Means and standard deviations of normalised signal densities of FN1 (**B**), CD44 (**E**) and S100A9 (**H**) against their corresponding α-tubulin bands. Comparison of abundance ratios for HGCA / LGCA as detected by LC–MS/MS and western blotting (WB) for FN1 (**C**), CD44 (**F**) and S100A9 (**I**)

To validate the LC–MS/MS results, western blotting (WB) was performed for representative selected proteins that had large differences in relative abundance with high statistical significance (Fig. 2). Densitometry was carried out on blots, and this method of semi-quantitation was compared with the LC–MS/MS quantification data for each individual sample.

FN1 was selected to validate the LC–MS/MS result in cell lines due to significant downregulation in HGCA cell lines relative to LGCA cell lines (Table 3). Densitometry revealed a reduction in HGCA-derived cell lines relative to LGCA-derived cell lines (Fig. 2B), with an intensity ratio for HGCA / LGCA of 0.52 (Fig. 2C), which was similar to the abundance ratio for LC–MS/MS (HGCA / LGCA = 0.35) (Table 3).

CD44 was selected due to its significant upregulation in HGCA-derived cell lines relative to LGCA cell lines (abundance ratio HGCA / LGCA = 2.27) (Table 3) and its relevance as a potential stem cell marker. WB confirmed this trend, with a strong signal detected in the 4 HGCA cell lines, and weaker bands seen in the 4 LGCA cell lines (Fig. 2D) at about 75 kDa. Densitometry revealed a normalised intensity ratio for HGCA / LGCA of 1.22 (Fig. 2F), but this difference was not statistically significant (Fig. 2E). However, the WB confirms the result seen in LC–MS/MS, in that CD44 was more abundant in the HGCA-derived cells than in the LGCA-derived cells.

S100A9 was selected because there was a sequential increase in abundance ratio for LGCA / NC (1.81), HGCA / LGCA (2.52) and HGCA / NC (4.86) in the tissue samples (Table 3). This was verified by WB using two LGCA and four HGCA tissues and their patient-matched NC tissue samples (LGNC1-2 and HGNC1-4, respectively). This showed that HGCA tissues had stronger bands than the LGCA tissues for S100A9 at about 13 kDa (Fig. 2G), and bands were faint or absent in the NC tissues. Densitometry revealed an HGCA / LGCA abundance ratio of 2.81 (Fig. 2I), confirming the upregulation detected by LC–MS/MS with a ratio of 2.52 (Fig. 2I, Table 3). In the cell lines, there was little to no signal detected in LGCA or HGCA cell lines (Additional File 12), which was consistent with the LC–MS/MS results (Table 3).

## Discussion

### DNA sequencing and proteomics validate tumour-derived cell lines

In order to assess the suitability of the tumour-derived primary cell lines as in vitro models of disease, DNA sequencing was performed to confirm that they carried over key mutations present in the parent tumour and did not diverge too far from the parent tissue by accumulating new mutations.

Inactivating mutations to the *APC* gene are the most common mutations in CRC [3, 4]. They lead to constitutive Wnt signalling, a process considered to be the initiating factor in CRC development [3]. Along with MMR genes such as *MSH2* and *MLH1*, *APC* can be used as a predictive marker of CRC development [3]. Of the 32 individual mutations to the APC gene detected across the 7 CA tissues, 26 were conserved in the tissue-derived cell lines. There were 4 instances of a mutation detected in only 1 out of 7 CA tissues being lost in the cell line derived from it, and one instance of a mutation arising in only 1 of the 7 cell lines but absent from all other samples, suggesting that these mutations are likely to be sequencing errors. There were two instances of a mutation being detected in both the tissue and cell line of only 1 patient, and these are assumed to be true low-frequency or passenger mutations.

*TP53* usually suffers from biallelic deactivation in CRC—one copy mutated and the other lost in a chromosomal deletion (17q), and this loss is associated with malignant transformation [3]. It has been suggested that CRC tumours with MMR defects usually retain a wild-type *TP53* [3], however the 4 samples in this cohort with the c.215 C > A *TP53* mutation also carried mutations in the *MSH2*, *MSH6* and/or *MLH1* genes, suggesting a hypermutated phenotype [4]. *MSH6* mutations in particular are prevalent in the hypermutated phenotype [4], and these mutations were very common in the LGCA samples (Additional File 3).

Interestingly, the LGCA tumours contained more mutations within these CRC-related genes than HGCA tumours; however, HGCA tumours had larger TMB scores (Additional File 4). This indicates that mutations in these CRC-related genes lead to CRC initiation, and that once the tumour is established and there are mutations present within the MMR genes, a range of other mutations arise that lead to progression of the tumour from LGCA to HGCA or get carried through as passengers.

The high degree of similarity between the tissues and cell lines, in terms of conservation of mutations in key CA-related genes and overlap of their proteomes, lends support to the idea that the cell lines are a reasonable representation of the parent tissue and a suitable in vitro model system. A recent study used a similar methodology to assess the conservation of mutations in primary cell lines derived from meningioma tissues [34].

The proteomes of the LGCA cell lines were less variable than those of the LGCA and HGCA tissues (Additional File 8), reflecting the heterogeneous mix of cell types in tissue, whereas the cell lines are comparatively homogenous. This aligned with the DNA sequencing data, which detected greater variability in terms of mutational burden between tissue samples than between cell lines. The tissue samples include muscle, blood vessels, immune cells and fat, and the process of tumourigenesis leads to large changes due to the loss of normal architecture and function as a dense bulk of tumour cells forms. Accordingly, the CA tissues exhibited significant decreases in proteins involved in muscle structure and contraction (CAV1, CAV2, ANK2, CNN1, TPM1, TPM2, MYL9, MYH11) that were not reflected in the cell lines. The downregulation of muscle system processes was also reported by Vasaikar et al*.* [23].

### CA tissue-derived primary cell lines reveal unique biological functions of tumours

Analysis of the cell lines revealed the upregulation of ferroptosis and interferon-gamma (IFN-g)-mediated signalling pathways in HGCA-derived cells relative to LGCA-derived cells (Fig. 1C, Additional File 11).

Ferroptosis is a recently recognised form of regulated cell death by iron-dependent lipid peroxidation that causes cellular membrane damage and the accumulation of reactive lipid hydroperoxides (known as lipid-ROS) to lethal levels [37]. Long-chain-fatty-acid-CoA ligase 4 (ACSL4) is an important modulator promoting ferroptosis via enhancing lipid peroxidation [38, 39]. ACSL4 catalyses the esterification of free fatty acids, preferentially polyunsaturated fatty acids (PUFAs), and incorporates esterified PUFAs into phospholipids within the cell membrane, creating substrates for lipoxygenases for lipid peroxidation. ACSL4 was significantly upregulated in HGCA cell lines relative to LGCA cells lines (Table 3), suggesting that these cells have an elevated potential to undergo ferroptosis (Additional File 11A). Induction of ferroptosis to target therapy-resistant CRC tumour cells has been proposed [38], with ACSL4 potentially representing a novel biomarker for treatment.

Ferroptosis can be antagonised by neutralising lipid-ROS by coupling the oxidation reaction of glutathione [37]. Glutamate-cysteine ligase (GCL), composed of catalytic (GCLC) and modifier (GCLM) subunits, is the rate-limiting enzyme in the glutathione biosynthesis pathway [40]. GCLM was significantly upregulated in HGCA cell lines relative to LGCA cell lines (Table 3), indicating that the HGCA cells have an elevated capacity for glutathione production to defend against ferroptosis. There is currently no established link between GCLM and CRC. this result suggests that inhibition of GCLM would allow ferroptosis to occur and lead to CRC cell death. GCL inhibition by buthionine sulfoximine has been demonstrated to induce ferroptosis in cultured pancreatic cell lines [41], and may be useful in targeting CRC cells when used in tandem with a GCLM inhibitor.

Cellular iron is another factor regulating ferroptosis via the Fenton reaction to produce lipid-ROS, and acts as a cofactor for lipoxygenases that catalyse lipid peroxidation [42]. When in circulation, iron forms a complex with transferrin, which binds the transferrin receptor protein-1 (TFRC) on the cell membrane to be taken up by the cell [42]. Excess iron can be stored as ferritin or exported from the cell. Ferroptosis can be triggered when there is an excess of iron stored within the cell or when iron uptake is increased [42]. TFRC was found to be significantly upregulated in the HGCA cell lines relative to the LGCA cell lines (Table 3), indicating increased capacity for iron uptake, possible over-accumulation of iron and subsequent ferroptosis. Furthermore, heme oxygenase 1 (HMOX1), which increases cellular iron levels by metabolising heme, was also significantly upregulated in these cells. HMOX1 has previously been shown to be expressed in CRC tissues and cell lines [43] and to promote erastin-induced ferroptosis [44, 45]. TFRC has not been previously linked with CRC but may be a useful biomarker.

In addition, there is evidence that p53 is involved in the regulation of ferroptosis. Xie et al. [46] reported that p53 loss increased the sensitivity of CRC cells to erastin-induced ferroptosis. This was due to the interaction between p53 and dipeptidyl-peptidase-4 (DPP4)—this complex translocates into the nucleus where DPP4 can act as a transcription cofactor. However, in *TP53* mutants where p53 is reduced or absent, DPP4 forms a complex with NOX1 to promote lipid peroxidation. *TP53* is one of the most commonly mutated genes in CRC, and at least one mutation was detected in both the tissues and cell lines from 5 of the 7 samples (Additional File 3). Therefore, in many CRCs there will be a reduction or loss of p53 and a heightened capacity for ferroptosis, supporting the proposal that ferroptosis induction could target CRC cells. All the above results together suggest that ferroptosis may be an effective target for cancer therapies for CRC.

IFN-g is a cytokine produced by immune cells in response to other cytokines or antigen stimulation to drive immune responses [47]. It signals via the IFN-g receptor, which is expressed by most, if not all, cell types. Binding of IFN-g to its receptor initiates JAK-STAT signalling, primarily through STAT1 which binds conserved DNA elements called INF-g activation sites to induce the transcription of interferon-stimulated genes [47]. The products of these genes regulate chemokine production, MHC molecules, antiviral and antibacterial factors, the function of regulators of metabolism, chromatin and transcription [47]. IFN-g upregulates the production of proteasomal subunits and the formation of immunoproteasomes, which produce peptides that bind more efficiently to MHC class I molecules [48]. In this way, IFN-g can increase the amount of antigen being presented to immune cells to build an immune response. The IFN-g-induced increase in immunoproteasomes and MHC class I components may allow for greater presentation of cancer antigens to help raise an anti-tumour immune response, and this might indicate a greater chance of immunotherapies being successful. IFN-g signalling was enriched in the HGCA-derived cell lines relative to LGCA-derived cell lines due to significant upregulation of guanylate-binding protein 1 (GBP1), vascular cell adhesion protein 1 (VCAM1), MCH class I components HLA-A and HLA-B, and CD44.

The LGCA tissues displayed decreased cell adhesion (FN1, VTN, VCAM, CEACAM5/6) relative to NC tissues (Fig. 1A, Additional File 9B). Conversely, HGCA-derived cell lines displayed upregulation of proteins related to adhesion (CAV1/3, VCAM1, CD44) and cytoskeletal binding (FN1, CDH2) relative to the LGCA-derived cell lines, as previously reported [23] (Fig. 1C, Additional File 11A). The observation of differences between the tissues and the cell lines in terms of adhesion and cytoskeletal binding are most likely due to the differences in the costs and benefits of adhesion between in vivo (tumour cell invasion and metastasis) and in vitro (growth as an adherent monolayer) conditions and may be influenced by a cell culture setting that lacks the 3D structural microenvironment present in vivo.

Interestingly, the data for FN1 and for MCH class I components from the tissues and cell lines were inconsistent. FN1 is one of many ECM proteins with aberrant expression in cancer, where it is associated with angiogenesis, invasion via matrix metalloproteinase activation, self-renewal, proliferation, and resistance to therapy, and high expression correlates with poor survival [49, 50]. One study demonstrated that silencing FN1 leads to increases in apoptosis-related proteins and reduced NF-kB, suggesting that FN1 overexpression in CA may aid tumour cells to evade apoptosis and to resist therapy by increasing NF-kB anti-apoptotic signalling [50]. Interestingly, in line with this finding, proteins involved in the positive regulation of NF-kB signalling such as ferroptosis-related proteins TFRC and HMOX1 were enriched in the HGCA-derived cell lines, despite FN1 being downregulated (Fig. 2, Additional File 11B), perhaps hinting at functional redundancy. MHC components HLA-A24, which preferentially presents tumour antigens with an aromatic residue at position 2 and a non-hydrophobic residue at the C-terminus, and HLA-B41, which displays self-peptides with Glu at position 2, were downregulated, whereas HLA-A69, associated with abnormal immune cell accumulation and suppression of the presentation of specific antigens, was upregulated; these factors all suggest a mechanism of immune avoidance by the cancer cells.

The Wnt signalling pathway is heavily implicated in cancer stem cell (CSC) function, and is considered the first pathway to be altered in CA development [3]. APC membrane recruitment protein 3 (AMER3), a positive Wnt signalling effector [51], was surprisingly downregulated in the cell lines and not detected in the tissues (Table 3, Additional Files 5 and 6). This may be because the Wnt signalling pathway is already constitutively activated in CA, making these enhancers redundant. Secreted frizzled-related protein 4 (SFRP4), which directly interacts with Wnt proteins and has been suggested as a marker of early-onset colon cancer [52], was overexpressed in CA tissues (Table 3). Furthermore, the cancer-associated scaffold protein syntenin-1 (SDCBP) is known to interact with Wnts [53] and is associated with colon CSC expansion, migration and chemoresistance [54]; it was upregulated in HGCA tissues and non-significantly increased in the HGCA cell lines (Table 3). CD44 is of particular interest in CRC as it is considered a CSC marker and its transcription is partially mediated through Wnt signalling [55, 56]. CD44 upregulation was observed in HGCA-derived cell lines relative to LGCA-derived cell lines (Fig. 2D–F; Table 3). CD44 may become a useful prognostic biomarker by aiding in tumour grading and estimating CSC presence. Structural maintenance of chromosomes protein 2 (SMC2) is involved in chromosome stability and DNA packaging as a component of the condensin complex [57–59]. The *SMC2* gene is a Wnt signalling target, and miRNA silencing of *SMC2* reduces intestinal tumour cell proliferation [59]. DNA supercoiling is vital to embryonic stem cell survival and SMC2 has been explored as a CSC-specific therapeutic target [58]. SMC2 displayed a fold-change increase of approximate 2.5 from LGCA to HGCA, with no detection in the NC tissues (Table 3), suggesting that its expression may be related to CA initiation and its upregulation related to progression.

The comparison of the proteomes of CA tissues with patient-matched tissue-derived primary cell lines provides unique insight. The cell lines, which are a purer population that retain the mutational signatures of the original tumour tissue, represents a unique and powerful method of analysing changes that are more relevant to the signalling within and between tumour cells without being overwhelmed by the large-scale changes occurring across the complexity of a tissue sample. For example, the HGCA cell lines revealed enrichment for IFN-g signalling and ferroptosis not seen in the comparison of NC, LGCA and HGCA tissues, which instead revealed less specific immune-related changes (e.g., “immune system process”, “leukocyte-mediated immunity”).

Overall, this suggests that within the tissue there are many unique physiological and structural changes occurring that are important aspects of the loss of normal function and the response of the body to the tumour, and the primary cell lines supplemented these findings by revealing changes in proteins involved in immune interactions and ferroptosis that went undetected in the tissues.

### Potential biomarker candidates and therapeutic drug targets for CA

This study has identified various proteins of interest that warrant further research as potential CA biomarkers, as well as validating some that have previously been identified (Table 3). Proteins such as ACE2, CD44, CNN1, FN1, HMOX1, MUC1 and MUC2, S100A8/S100A9, SDCBP, SMC2, and SFRP4 were significantly differentially expressed and have been previously reported in CA. However, a range of new protein biomarker candidates for CA, including ACSL4, AMER3, ANK2, EXOSC1, EXOSC6, GCLM, and TFRC, were found in this study, particularly in the tissue-derived cell lines.

ACE2 is a component of the renin-angiotensin system (RAS) that catalyses the production of Mas receptor (MasR) ligands Ang1-9 and Ang1-7 [60]. MasR signalling reduces inflammation and susceptibility to cardiovascular diseases [61]. ACE2 loss has therefore been predicted to be a marker of poor prognosis in CA [62, 63]. However, it was detected in only one NC tissue but found at high abundance in all LGCA and HGCA tissues (Additional Files 5 and 6). The role of ACE2 and the MasR in cancer is still unclear, with reports of decreased ACE2 in breast and pancreatic cancers [63], but contradictory reports of ACE2 and MasR overexpression in CRC, and MasR-mediated cancer cell migration in renal cell carcinoma [63, 64]. The Human Protein Atlas supports the finding of elevated ACE2 levels in CRC and suggests that ACE2 is overexpressed in renal, pancreatic and liver cancers (https://www.proteinatlas.org/ENSG00000130234-ACE2/pathology). It is possible that ACE2 plays a role in CRC outside of the RAS, or that the outcomes of MasR signalling depend on their physiological context. The importance of ACE2 in CRC deserves further research.

Ankyrins are adapter proteins that organise integral membrane proteins including cell junction proteins and cell adhesion molecules, ion channels and transporters by anchoring them to the spectrin-based membrane skeleton within the cell [65–67]. ANK2 was present in the network of proteins significantly downregulated in LGCA tissues relative to NC. In this network, ANK2 was linked with CACNA1D, a subunit of voltage-dependent calcium channels that facilitate the movement of calcium ions into cells to allow calcium-dependent processes to occur, including muscle contraction. The loss of ANK2 could reflect the physiological changes that occur in the gut during cancer development, including the loss of muscle function in the tumour. Alternatively, low levels of ANK2 would lead to a reduced capacity to anchor other integral membrane proteins to the spectrin skeleton, preventing the collection of multiprotein complexes. This may cause aberrant signalling, either due to members of signalling pathways being unable to group efficiently within the membrane, or alternatively, by allowing proteins that require anchoring to isolate them from a signalling complex to move freely through the membrane and interact with their partners. ANK2 has not yet been associated with CA in the literature and may represent a new biomarker for CA development. Our finding that ANK2 is significantly downregulated in LGCA tissues relative to matched NC suggests that impaired ability to localise and stabilise other transmembrane proteins and interact with adhesion molecules may be implicated in CA progression.

The CA tissues displayed increased levels of preribosomal components (CIRH1A, NIP7, BYSL) and RNA processing members (EXOSC1, EXOSC6). U3 small nucleolar RNA-associated protein 4 (CIRH1A/UTP4) strongly promotes CA cell proliferation and reduces apoptosis [68]; it was not detected in NC tissues but was present in CA tissues, and may represent a potential biomarker for CA initiation and a therapeutic target to reduce tumour proliferation. Exosome complex component 6 (EXOSC6) is a non-catalytic component of the RNA exosome complex that performs RNA processing and degradation [69]. Based on its low expression levels in the NC and stepwise increases in abundance in LGCA and HGCA tissues, it may be a candidate for further research as a potential marker of CA progression.

Calponin-1 (CNN1) regulates smooth muscle contraction by binding actin, calmodulin and tropomyosin [70, 71]. Downregulation of CNN1 leads to a loss of membrane integrity in smooth muscle, the uterus and peritoneum, causing blood vessels to become leaky and allowing cancer cell intravasation [72, 73]. CNN1 is considered cancer-suppressive, and indeed it is known to be downregulated in cancers [72, 74]. It is thought to be a better marker than αSMA for smooth muscle cell differentiation [74]. Tumour vasculature has an immature phenotype characterised by incomplete pericyte coverage, irregular shapes and growth patterns, and permeable membranes, which may in part be due to CNN1 loss [74]. This allows tumour cells to enter vessels and metastasise [74]. In the CA tissues and tissue-derived cell lines, CNN1 levels fell in a stepwise manner from NC to LGCA and LGCA to HGCA. Collectively, CNN1 loss may indicate the initiation of CA and it may represent a useful predictor of invasion and metastatic potential in CA.

S100A8 (MRP8) and S100A9 (MRP14) are commonly found as a heterodimer called calprotectin that binds Ca^2+^ and Zn^2+^ ions and plays an important role in inflammation caused by infection, autoimmunity or metabolic diseases [75]. Upregulation of S100A8 and S100A9 induces chemotaxis of leukocytes, cytokine release and apoptosis during inflammation, a key aspect of CRC [75]. Accordingly, other studies have reported significant overexpression in CRC [18, 23]. Similarly, the abundance of S100A8 and S100A9 here was found to increase in a stepwise manner from NC to LGCA and LGCA to HGCA (Table 3; Fig. 2G–I). Unsurprisingly, they were not detected in the cell lines because they are not produced by the cancer cells themselves, but by neutrophils recruited to the site of the tumour. The detection in the blood of S100A8/S100A9 released by neutrophils in the tumour microenvironment suggests they could be utilised as a serum biomarker for diagnosis or prognosis of CA.

Functional assays, including miRNA silencing or CRISPR knock-outs, would clarify the roles of these potential CRC biomarkers.

## Conclusions

Proteomic analysis revealed that when compared to their NC controls, CA tissues displayed increases in preribosome function and RNA processing, and loss of normal muscle function. The alignment of DNA sequencing and LC–MS/MS data from both the CA tissues and CA-derived cell lines and with published data from other CRC proteogenomic studies suggests that patient-derived cell lines are a useful in vitro model for CA tumours, although they are not a complete reflection of the complex situation within CA tissues. The key differences between the two sample types included the global physiological changes such as to muscle structure and drug metabolism that were only seen in the tissue samples, and an increase in IFN-g and ferroptosis-related proteins in HGCA-derived cell lines. This study has highlighted a range of potential CRC protein biomarkers and drug targets including novel markers such as ferroptosis-related proteins ACSL4, TFRC and GCLM, membrane anchor protein ANK2, RNA exosome complex components EXOSC1 and EXOSC6, and Wnt effector AMER3. Furthermore, it has validated other CA-related proteins recently reported in the literature, including renin-angiotensin system component ACE2, membrane protein CNN1, and calprotectin components S100A8 and S100A9.

## Supplementary Information

**Additional file 1**. Patient demographics, pathology and biochemical characteristics.

**Additional file 2**. Representative images of FFPE NC and CA tissues. (A) Normal colon, (B) LGCA tumour, (C) HGCA tumour. IHC staining for c-MYC was performed. Original magnification: 200x.

**Additional file 3**. Mutations to CA-related genes in FFPE CA tissues and CA-derived primary cell lines.

**Additional file 4**. Summary of TML assay results for CA tissues and CA-derived primary cell lines.

**Additional file 5**. Lists of all proteins identified in NC and CA tissues and proteins with significantly differential abundance between tissue samples. Legend is available on Sheet 1. Abbreviations: LG = low-grade colon adenocarcinoma, HG = high-grade colon adenocarcinoma, NC = normal colon.

**Additional file 6**. Lists of all proteins identified in CA tissue-derived primary cell lines and proteins with significantly differential abundance in HGCA cell lines relative to LGCA cell lines. Legend is available on Sheet 1. Abbreviations: LG = low-grade colon adenocarcinoma, HG = high-grade colon adenocarcinoma, NC = normal colon.

**Additional file 7**. List of proteins found in both the tissue samples and the primary cell lines. Abbreviations: LG = low-grade colon adenocarcinoma, HG = high-grade colon adenocarcinoma, NC = normal colon.

**Additional file 8**. Comparison of proteins identified in biological replicates for each condition. Abbreviations: LG = low-grade colon adenocarcinoma, HG = high-grade colon adenocarcinoma, NC = normal colon, Cells = Cell lines, T = Tumour tissue. The numbers in the graph titles denote the numbering of patients, with total number of proteins identified in each sample listed in brackets.

**Additional file 9**. Analysis of proteins with significantly differential expression in LGCA tissues compared to NC tissues. A, Proteins with significantly increased abundance with a moderate confidence level (0.4), with GO terms or KEGG or Reactome pathways of interest coloured as follows: Red – sterol biosynthesis; Blue – cholesterol biosynthesis; Green – exosome (rnase complex); Yellow – preribosome. B, Proteins with significantly decreased abundance with a moderate confidence level (0.4), with GO terms or KEGG or Reactome pathways of interest coloured as follows: Red – regulation of cytoskeleton organization; Blue – cellular component assembly involved in morphogenesis; Light Green – muscle system process; Yellow – caveolar macromolecular signalling complex; Pink – cytoskeleton organization; Dark Green – myofibril assembly; Teal – actin-mediated cell contraction; Orange – plasma membrane organization; Purple – actomyosin structure organization; Brown – cellular component morphogenesis.

**Additional file 10**. Analysis of proteins with significantly differential expression in HGCA tissues compared to LGCA tissues. A, Proteins with significantly increased abundance with a medium confidence level (0.4), with GO terms or KEGG or Reactome pathways of interest coloured as follows: Red – detoxification; Blue – cellular zinc ion homeostasis; Light Green – regulation of growth; Yellow – immune response; Pink – immune system process; Teal – neutrophil degranulation; Orange – positive regulation of cell growth; Purple – leukocyte mediated immunity; Brown – myeloid leukocyte activation. B, Proteins with significantly decreased abundance with a medium confidence level (0.4), with GO terms or KEGG or Reactome pathways of interest coloured as follows: Red – nitrogen metabolism; Blue – tyrosine metabolism; Yellow – drug metabolism cytochrome P450.

**Additional file 11**. Analysis of proteins with significantly differential expression in HGCA cell lines compared to LGCA cell lines. A, Proteins with significantly increased abundance with a medium confidence level (0.4), with GO terms or KEGG or Reactome pathways of interest coloured as follows: Red – IFN-g-mediated signalling pathway; Blue – ferroptosis; Yellow – growth plate cartilage morphogenesis; Light Green – negative regulation of leukocyte mediated immunity; Pink – response to IFN-g; Teal – regulation of i-kappab kinase/nf-kappab signaling; Orange – cellular response to chemical stress; Dark Green – cytokine-mediated signaling pathway. B, Proteins with significantly decreased abundance with a medium confidence level (0.4), with GO terms or KEGG or Reactome pathways of interest coloured as follows: Red – MHC class I protein complex; Blue – filopodium membrane; Green – dystrophin-associated glycoprotein complex; Yellow – cell adhesion; Pink – cell junction; Orange – plasma membrane protein complex.

**Additional file 12**. Western blotting for S100A9 protein extracted from CA tissue-derived primary cell lines. Fluorescent signals for S100A9 (A, red) and the loading control α-tubulin (A, green). Means and standard deviations of normalised signal densities of S100A9 (B) against their corresponding α-tubulin bands. Comparison of abundance ratios for HGCA / LGCA as detected by LC–MS/MS and western blotting (WB) for S100A9. Tonsil tissue was used as a positive control. Abbreviations: LG = low-grade colon adenocarcinoma, HG = high-grade colon adenocarcinoma.

## Data Availability

Mass spectral RAW files and Proteome Discoverer (version 2.4, ThermoFisher Scientific) result files are available on ProteomeXchange Consortium [35] via the PRIDE (36) partner repository with the dataset identifier PXD024449.

## Abbreviations

CA: Colon adenocarcinoma
CRC: Colorectal cancer
CSC: Cancer stem cell
ECM: Extracellular matrix
EMT: Epithelial-to-mesenchymal transition
FFPE: Formalin-fixed paraffin-embedded
GO: Gene ontology
HDC: High-energy collision-induced dissociation
HGCA: High-grade colon adenocarcinoma
IHC: Immunohistochemical
KEGG: Kyoto encyclopedia of genes and genomes
LFQ: Label-free quantitation
LGCA: Low-grade colon adenocarcinoma
MMR: Mismatch repair
MSI: Microsatellite instability
NC: Normal colon
PD: Proteome discoverer
RAS: Renin-angiotensin system
TMB: Tumour mutational burden
WB: Western blotting
