# Supplementary material for: Proteomic analysis of low- and high-grade human colon adenocarcinoma tissues and tissue-derived primary cell lines reveals unique biological functions of tumours and new protein biomarker candidates
Source: Clin Proteomics. 2022 Jul 16;19:27. doi: 10.1186/s12014-022-09364-y (PMC9287856; doi:10.1186/s12014-022-09364-y)
Supplement: Supplementary file 1 — Additional file 1. Patient demographics, pathology and biochemical characteristics. [file 12014_2022_9364_MOESM1_ESM.docx]

**Additional File 1: Patient demographics, pathology and biochemical characteristics.**

| **Case** | **Age** | **Gender and ethnicity** | **TNM stage** | **Lab report and**  **genetic & molecular analyses** | **Comorbidity** | **Medication** |
| --- | --- | --- | --- | --- | --- | --- |
| **LG1** | 53 | Male,  NZ Euro | pT3 N1a Mx | Irregular cribriform glands; pleomorphic columnar cells. Normal for MLH1, MSH2, MSH6, PMS2. | Yes, not specified | Cilazapril |
| **LG2** | 67 | Male,  NZ Euro | pT3 pN1a+ Mx | Several small foci of high grade noted. | Yes, not specified | Unknown |
| **LG3** | 60 | Female,  NZ Euro | T3 N2a Mx | Spread to pericolic fat but not invasive though muscularis. MLH1, PMS2, MSH6, MSH2 normal (positive). | Yes, COPD, hyperthyroid, benign papillary mesothelioma | Carbimazole, Salbutamol, Striverdi- respimat |
| **LG4** | 73 | Unknown, NZ Euro | T3 N1b Mx | 1/32 lymph nodes, extramural tumour deposits; perineural invasion. MLH1, PMS2, MSH6, MSH2 normal (positive). | Unknown | Unknown |
| **HG1** | 50 | Male,  NZ Euro | pT3 N0 Mx | No records – surgery performed at private hospital. | Metastasis to liver & lung since recruitment | Unknown |
| **HG2** | 81 | Female,  NZ Euro | Absent from report | Moderately differentiated adenocarcinoma, no lymph nodes (0/7). | Unknown | Unknown |
| **HG3** | 74 | Female,  Indian | T3 N1 Mx | Tumour invades through muscularis propria into the serosa. | Unknown | Unknown |
| **HG4** | 70 | Male,  NZ Euro | pT4b N2a Mx | Invades through muscularis propria. MLH1, PMS2, MSH6, MSH2 normal (positive). | Unknown | Unknown |

Abbreviations: NZ Euro = New Zealand European; LG = low-grade colon adenocarcinoma; HG = high-grade colon adenocarcinoma.
