# Supplementary material for: Proteomic analysis of low- and high-grade human colon adenocarcinoma tissues and tissue-derived primary cell lines reveals unique biological functions of tumours and new protein biomarker candidates
Source: Clin Proteomics. 2022 Jul 16;19:27. doi: 10.1186/s12014-022-09364-y (PMC9287856; doi:10.1186/s12014-022-09364-y)

**Additional File 2: Representative images of formalin-fixed paraffin-embedded NC and CA tissues.** (A) Normal colon, (B) LGCA tumour, (C) HGCA tumour. IHC staining for c-MYC was performed. Original magnification: 200x.


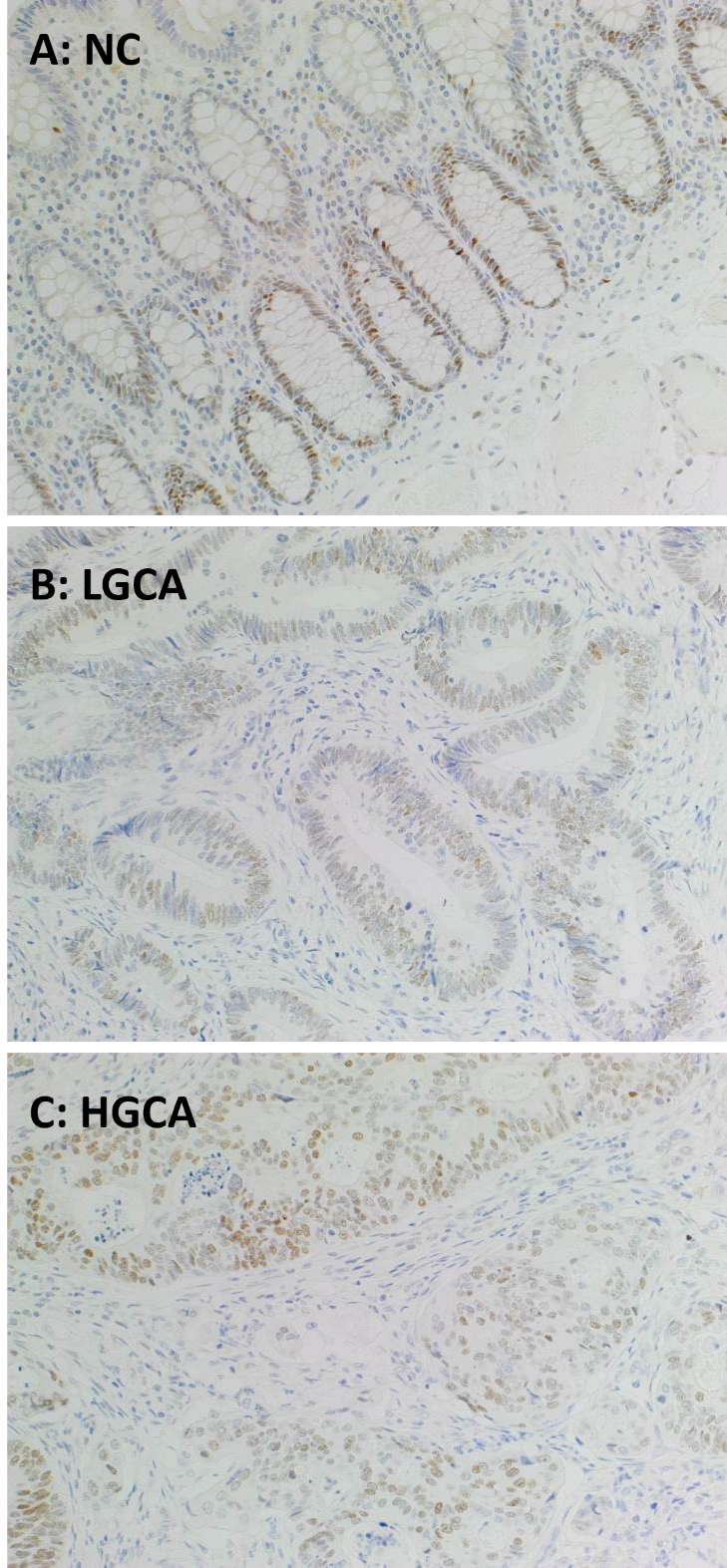

Supplement: Supplementary file 2 — Additional file 2. Representative images of FFPE NC and CA tissues. (A) Normal colon, (B) LGCA tumour, (C) HGCA tumour. IHC staining for c-MYC was performed. Original magnification: 200x. [file 12014_2022_9364_MOESM2_ESM.docx]
