# Supplementary material for: Proteomic analysis of low- and high-grade human colon adenocarcinoma tissues and tissue-derived primary cell lines reveals unique biological functions of tumours and new protein biomarker candidates
Source: Clin Proteomics. 2022 Jul 16;19:27. doi: 10.1186/s12014-022-09364-y (PMC9287856; doi:10.1186/s12014-022-09364-y)
Supplement: Supplementary file 3 — Additional file 3. Mutations to CA-related genes in FFPE CA tissues and CA-derived primary cell lines. [file 12014_2022_9364_MOESM3_ESM.docx]

**Additional File 3: Mutations to CA-related genes in FFPE CA tissues and CA-derived primary cell lines.**

| **Gene** | **Mutation** | | **LGCA1** | **LGCA2** | **LGCA3** | **HGCA1** | **HGCA2** | **HGCA3** | **HGCA4** |
| --- | --- | --- | --- | --- | --- | --- | --- | --- | --- |
| ***APC*** | c.646 C>T | **Tissue** | - | - | 🗸 | - | - | - | - |
|  |  | **Cells** | - | - | - | - | - | - | - |
|  | c.688_689 ins ACTTC | **Tissue** | - | - | 🗸 | - | - | - | - |
|  |  | **Cells** | - | - | - | - | - | - | - |
|  | c.1458 T>C | **Tissue** | 🗸 | 🗸 | 🗸 | 🗸 | - | 🗸 | 🗸 |
|  |  | **Cells** | 🗸 | 🗸 | 🗸 | 🗸 | 🗸 | 🗸 | - |
|  | c.1743+19 A>G | **Tissue** | - | 🗸 | - | - | - | - | - |
|  |  | **Cells** | - | 🗸 | - | - | - | - | - |
|  | c.1476 C>G | **Tissue** | - | - | - | - | - | - | - |
|  |  | **Cells** | - | - | - | 🗸 | - | - | - |
|  | c.3871 C>T | **Tissue** | - | 🗸 | - | - | - | - | - |
|  |  | **Cells** | - | - | - | - | - | - | - |
|  | c.4118 del C | **Tissue** | - | - | - | 🗸 | - | - | - |
|  |  | **Cells** | - | - | - | - | - | - | - |
|  | c.4479 G>A | **Tissue** | 🗸 | 🗸 | 🗸 | 🗸 | - | - | - |
|  |  | **Cells** | 🗸 | 🗸 | 🗸 | 🗸 | 🗸 | 🗸 | - |
|  | c.5034 G>A | **Tissue** | 🗸 | 🗸 | 🗸 | 🗸 | 🗸 | 🗸 | 🗸 |
|  |  | **Cells** | 🗸 | 🗸 | 🗸 | 🗸 | 🗸 | 🗸 | 🗸 |
|  | c.5268 T>G | **Tissue** | - | 🗸 | 🗸 | 🗸 | - | 🗸 | 🗸 |
|  |  | **Cells** | 🗸 | 🗸 | 🗸 | 🗸 | 🗸 | 🗸 | - |
|  | c.5880 G>A | **Tissue** | - | 🗸 | 🗸 | 🗸 | - | 🗸 | 🗸 |
|  |  | **Cells** | 🗸 | 🗸 | 🗸 | 🗸 | 🗸 | 🗸 | - |
|  | c.7201 C>T | **Tissue** | - | 🗸 | - | - | - | - | - |
|  |  | **Cells** | - | 🗸 | - | - | - | - | - |
| ***TP53*** | c.215 C>A | **Tissue** | 🗸 | 🗸 | 🗸 | 🗸 | - | - | - |
|  |  | **Cells** | 🗸 | 🗸 | 🗸 | 🗸 | - | - | - |
|  | c.391 A>T | **Tissue** | 🗸 | - | - | - | - | - | - |
|  |  | **Cells** | - | - | - | - | - | - | - |
|  | c.524 C>T | **Tissue** | - | - | - | 🗸 | - | - | - |
|  |  | **Cells** | - | - | - | - | - | - | - |
|  | c.538 G>T | **Tissue** | - | 🗸 | - | - | - | - | - |
|  |  | **Cells** | - | - | - | - | - | - | - |
|  | c.754 del C | **Tissue** | - | - | 🗸 | - | - | - | - |
|  |  | **Cells** | - | - | - | - | - | - | - |
|  | c.844 G>A | **Tissue** | - | - | - | - | 🗸 | - | - |
|  |  | **Cells** | - | - | - | - | - | - | - |
|  | c.919+1 G>A | **Tissue** | - | - | - | - | - | - | 🗸 |
|  |  | **Cells** | - | - | - | - | - | - | 🗸 |
| ***KRAS*** | c.483 C>T | **Tissue** | 🗸 | 🗸 | 🗸 | 🗸 | - | - | - |
|  |  | **Cells** | 🗸 | 🗸 | 🗸 | 🗸 | 🗸 | 🗸 | - |
|  | c.*5598 A>G | **Tissue** | - | - | - | - | - | - | - |
|  |  | **Cells** | - | - | - | - | 🗸 | - | - |
| ***BRAF*** | c.980+27 C>T | **Tissue** | 🗸 | 🗸 | - | - | - | - | - |
|  |  | **Cells** | 🗸 | 🗸 | - | - | - | - | - |
|  | c.1518-48 G>A | **Tissue** | 🗸 | 🗸 | - | - | - | - | - |
|  |  | **Cells** | 🗸 | 🗸 | - | - | - | - | - |
| ***MSH2*** | c.211+9 C>G | **Tissue** | 🗸 | - | 🗸 | - | - | - | - |
|  |  | **Cells** | 🗸 | - | 🗸 | - | - | - | - |
|  | c.2006-6 T>C | **Tissue** | - | - | 🗸 | - | - | - | - |
|  |  | **Cells** | - | - | 🗸 | - | - | - | - |
| ***MSH6*** | c.186 C>A | **Tissue** | - | - | - | - | - | - | 🗸 |
|  |  | **Cells** | - | - | - | - | - | - | - |
|  | (with FBXO11:) c.3647-70_3647-54 del TTTTTGTTTTAATTCCT, c.*1985 AGGAATTAAAACAAAAAT>T | **Tissue** | - | 🗸 | - | - | - | 🗸 | - |
|  |  | **Cells** | - | 🗸 | - | - | - | 🗸 | - |
|  | c.116 G>A | **Tissue** | - | 🗸 | 🗸 | - | - | - | - |
|  |  | **Cells** | - | 🗸 | 🗸 | - | - | - | - |
|  | c.540 T>C | **Tissue** | - | 🗸 | - | - | - | - | - |
|  |  | **Cells** | - | 🗸 | - | - | - | 🗸 | - |
|  | c.642 C>T | **Tissue** | - | 🗸 | - | - | - | - | - |
|  |  | **Cells** | - | 🗸 | - | - | - | 🗸 | - |
|  | c.1186 C>G | **Tissue** | - | - | - | 🗸 | - | - | - |
|  |  | **Cells** | - | - | - | 🗸 | - | - | - |
|  | c.3306 T>A | **Tissue** | - | - | 🗸 | - | - | - | - |
|  |  | **Cells** | - | - | 🗸 | - | - | - | - |
|  | c.3438+14 A>T | **Tissue** | 🗸 | - | - | 🗸 | - | - | - |
|  |  | **Cells** | 🗸 | - | - | 🗸 | - | - | - |
|  | c.3646+29_3646+32 del CTAT | **Tissue** | 🗸 | 🗸 | 🗸 | 🗸 | - | - | - |
|  |  | **Cells** | 🗸 | 🗸 | 🗸 | 🗸 | 🗸 | 🗸 | - |
|  | c.3646+35_3646+40 indel T | **Tissue** | - | - | - | 🗸 | - | - | - |
|  |  | **Cells** | - | - | 🗸 | 🗸 | - | - | - |
|  | c.3646+91 T>C | **Tissue** | 🗸 | 🗸 | 🗸 | 🗸 | - | - | - |
|  |  | **Cells** | 🗸 | 🗸 | 🗸 | 🗸 | 🗸 | - | - |
| ***MLH1*** | c.655 A>G | **Tissue** | 🗸 | - | - | - | - | 🗸 | - |
|  |  | **Cells** | 🗸 | - | - | - | - | 🗸 | - |
|  | c.1558+14 G>A | **Tissue** | - | 🗸 | - | - | 🗸 | - | 🗸 |
|  |  | **Cells** | - | 🗸 | - | - | 🗸 | - | 🗸 |
|  | c.1668-19 A>G | **Tissue** | 🗸 | 🗸 | - | - | - | - | - |
|  |  | **Cells** | 🗸 | 🗸 | - | - | - | 🗸 | - |
| ***PIK3CA*** | c.1060-17 C>A | **Tissue** | - | 🗸 | - | - | - | 🗸 | 🗸 |
|  |  | **Cells** | 🗸 | 🗸 | - | - | 🗸 | 🗸 | - |
|  | c.1173 A>G | **Tissue** | - | - | - | - | - | - | - |
|  |  | **Cells** | - | - | - | - | - | 🗸 | - |
|  | c.2295-57 C>G | **Tissue** | 🗸 | 🗸 | - | - | - | - | - |
|  |  | **Cells** | 🗸 | 🗸 | - | - | 🗸 | 🗸 | - |
|  | c.2416+67 A>G | **Tissue** | - | - | 🗸 | - | - | - | - |
|  |  | **Cells** | - | - | 🗸 | - | - | - | - |
| ***FBXW7*** | c.1855+67 G>A | **Tissue** | 🗸 | - | - | - | - | - | - |
|  |  | **Cells** | 🗸 | - | - | - | - | - | - |
|  | c.1972 G>A | **Tissue** | - | - | - | - | - | - | 🗸 |
|  |  | **Cells** | - | - | - | - | - | - | 🗸 |
| **% mutations shared by tissues and cells)** | | | 78.9% | 91.3% | 77.8% | 80.0% | 15.4% | 46.7% | 44.4% |

Mutations detected in the FFPE CA tissues and CA-derived cells for 9 CA-related genes are displayed. Presence (🗸) or absence (-) of mutations are indicated. Green font is used for mutations that were detected in both the CA tissue and CA-derived cells from one patient, and red font for mutations only found in one sample type from each patient. Mutation types are substitutions (>), deletions (del), insertions (ins) and insertion + deletion mutations (indel). The percentage of mutations shared by the CA tissue and the CA-derived cell line from each patient were calculated by dividing the number of mutations shared by tissues and cells (green ticks) by the total number of mutations across the tissues and/or the cells (green and red ticks) and are displayed at the bottom of each column.
