# Supplementary material for: Proteomic analysis of low- and high-grade human colon adenocarcinoma tissues and tissue-derived primary cell lines reveals unique biological functions of tumours and new protein biomarker candidates
Source: Clin Proteomics. 2022 Jul 16;19:27. doi: 10.1186/s12014-022-09364-y (PMC9287856; doi:10.1186/s12014-022-09364-y)
Supplement: Supplementary file 4 — Additional file 4. Summary of TML assay results for CA tissues and CA-derived primary cell lines. [file 12014_2022_9364_MOESM4_ESM.docx]

**Additional File 4: Summary of TML assay results for CA tissues and CA-derived primary cell lines.**

| **Samples** | **TMB (mutations/Mb)** | | **Total variants** | | | **Impactful variants** | | |
| --- | --- | --- | --- | --- | --- | --- | --- | --- |
|  | **Cells** | **Tissues** | **Cells** | **Tissues** | **Shared** | **Cells** | **Tissues** | **Shared** |
| LGCA1 | 3.36 | 4.63 | 154 | 40 | 1856 | 23 | 6 | 263 |
|  |  |  | 2050 | | | 292 | | |
| LGCA2 | 1.67 | 5.92 | 44 | 63 | 1986 | 4 | 9 | 291 |
|  |  |  | 2093 | | | 304 | | |
| LGCA3 | 1.67 | 6.01 | 58 | 40 | 1925 | 5 | 4 | 272 |
|  |  |  | 2023 | | | 281 | | |
| HGCA1 | 2.51 | 7.75 | 64 | 163 | 1932 | 7 | 27 | 278 |
|  |  |  | 2159 | | | 312 | | |
| HGCA2 | 2.51 | 10.49 | 95 | 519 | 1445 | 17 | 97 | 201 |
|  |  |  | 2059 | | | 315 | | |
| HGCA3 | 1.68 | 38.65 | 165 | 477 | 1540 | 24 | 65 | 230 |
|  |  |  | 2182 | | | 319 | | |
| HGCA4 | 20.69 | 17.67 | 83 | 124 | 1436 | 24 | 53 | 189 |
|  |  |  | 1643 | | | 266 | | |

Tumour mutational burden (TMB) score, the total number of variants within the 409 TML assay genes and the number of variants which impact the gene product (i.e. missense, nonsense, etc.) are displayed. For each CA sample, the number of variants unique to the cells and to the tissues and those shared by both are reported, with the total number of variants listed directly below.
