# Supplementary figures and images for: Proteomic analysis of low- and high-grade human colon adenocarcinoma tissues and tissue-derived primary cell lines reveals unique biological functions of tumours and new protein biomarker candidates
Source: Clin Proteomics. 2022 Jul 16;19:27. doi: 10.1186/s12014-022-09364-y (PMC9287856; doi:10.1186/s12014-022-09364-y)

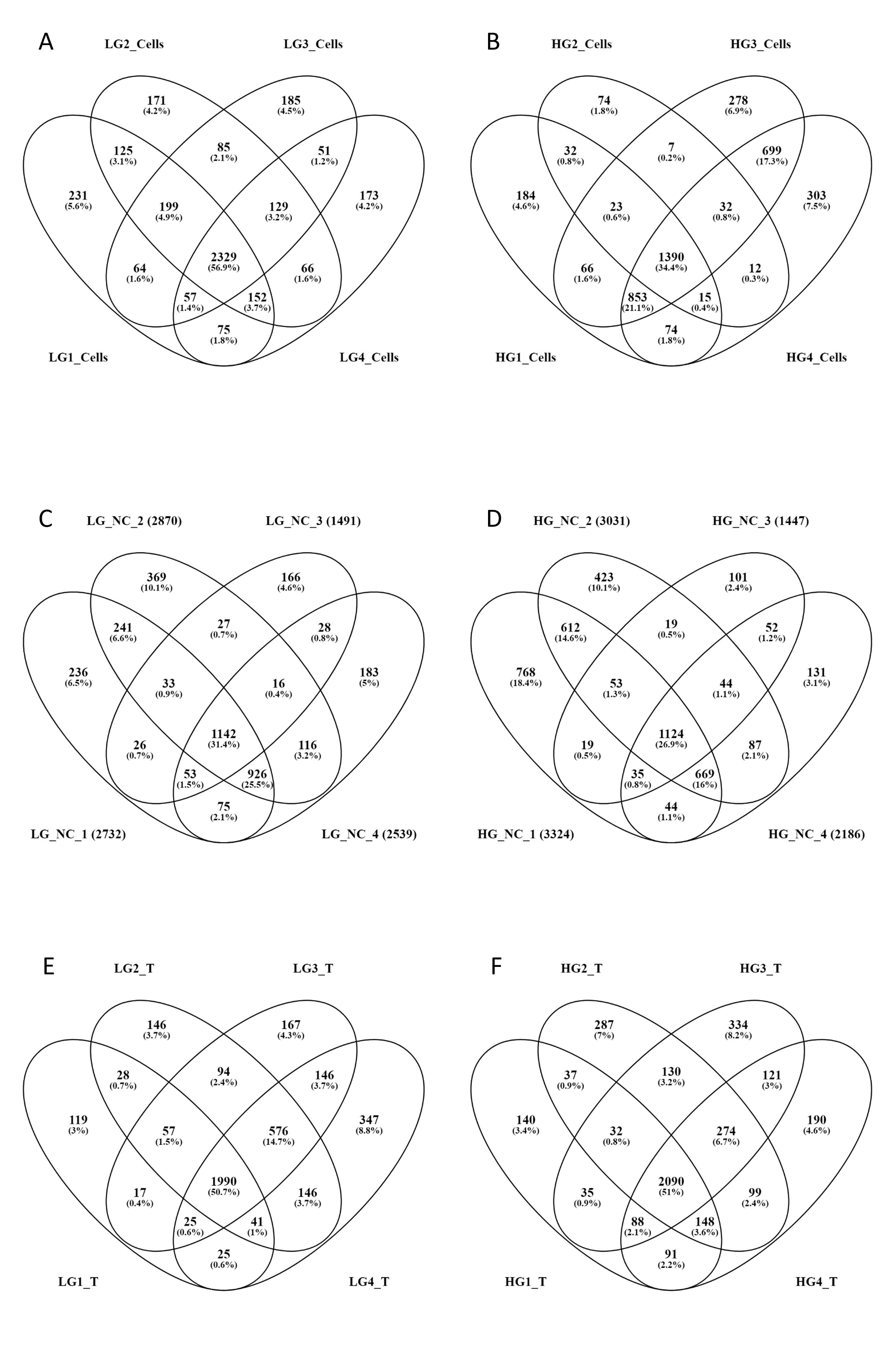

Supplement: Supplementary file 8 — Additional file 8. Comparison of proteins identified in biological replicates for each condition. Abbreviations: LG = low-grade colon adenocarcinoma, HG = high-grade colon adenocarcinoma, NC = normal colon, Cells = Cell lines, T = Tumour tissue. The numbers in the graph titles denote the numbering of patients, with total number of proteins identified in each sample listed in brackets. [file 12014_2022_9364_MOESM8_ESM.tif]

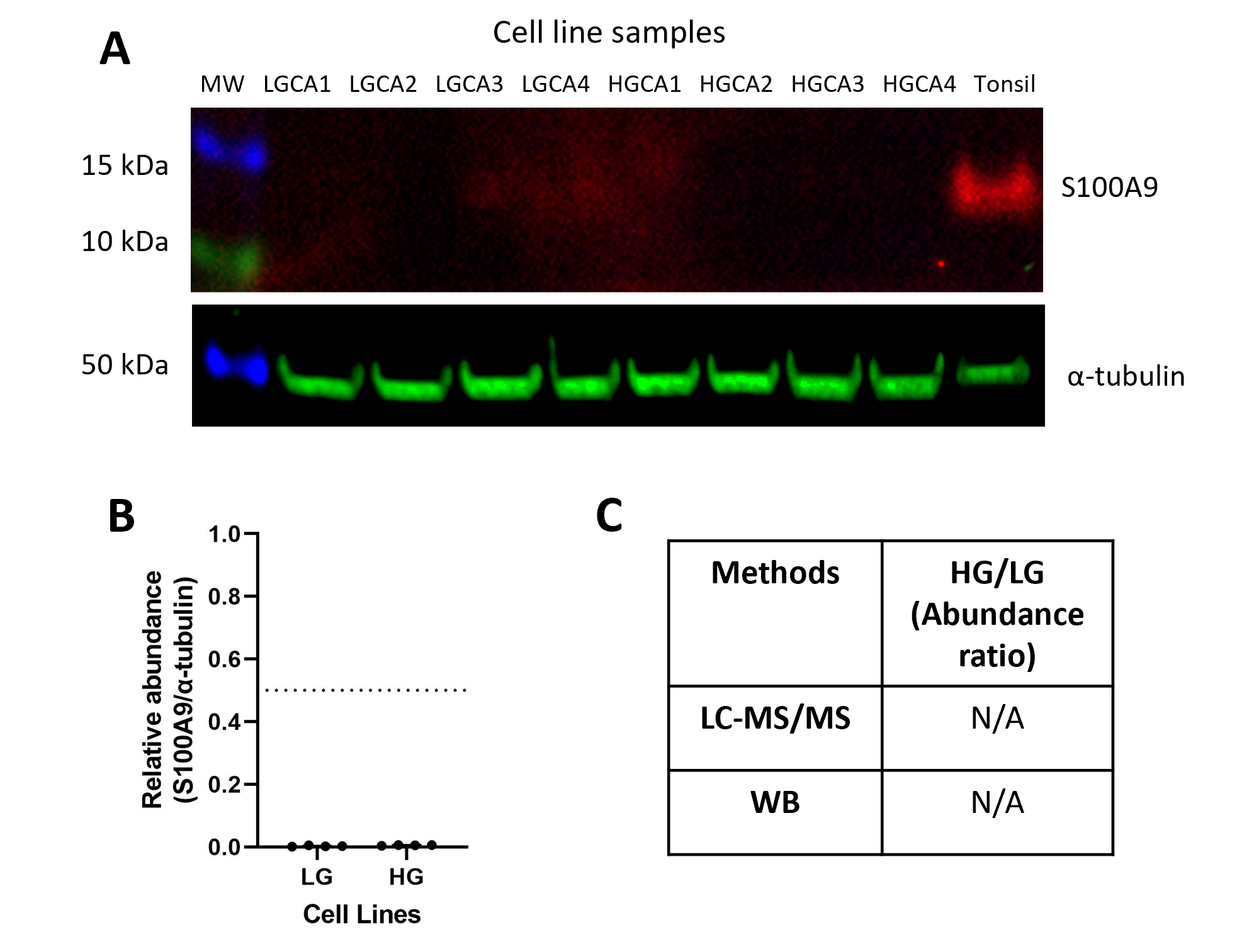

Supplement: Supplementary file 12 — Additional file 12. Western blotting for S100A9 protein extracted from CA tissue-derived primary cell lines. Fluorescent signals for S100A9 (A, red) and the loading control α-tubulin (A, green). Means and standard deviations of normalised signal densities of S100A9 (B) against their corresponding α-tubulin bands. Comparison of abundance ratios for HGCA / LGCA as detected by LC–MS/MS and western blotting (WB) for S100A9. Tonsil tissue was used as a positive control. Abbreviations: LG = low-grade colon adenocarcinoma, HG = high-grade colon adenocarcinoma. [file 12014_2022_9364_MOESM12_ESM.tif]
