# Supplementary material for: Proteomic analysis of low- and high-grade human colon adenocarcinoma tissues and tissue-derived primary cell lines reveals unique biological functions of tumours and new protein biomarker candidates
Source: Clin Proteomics. 2022 Jul 16;19:27. doi: 10.1186/s12014-022-09364-y (PMC9287856; doi:10.1186/s12014-022-09364-y)
Supplement: Supplementary file 9 — Additional file 9. Analysis of proteins with significantly differential expression in LGCA tissues compared to NC tissues. A, Proteins with significantly increased abundance with a moderate confidence level (0.4), with GO terms or KEGG or Reactome pathways of interest coloured as follows: Red – sterol biosynthesis; Blue – cholesterol biosynthesis; Green – exosome (rnase complex); Yellow – preribosome. B, Proteins with significantly decreased abundance with a moderate confidence level (0.4), with GO terms or KEGG or Reactome pathways of interest coloured as follows: Red – regulation of cytoskeleton organization; Blue – cellular component assembly involved in morphogenesis; Light Green – muscle system process; Yellow – caveolar macromolecular signalling complex; Pink – cytoskeleton organization; Dark Green – myofibril assembly; Teal – actin-mediated cell contraction; Orange – plasma membrane organization; Purple – actomyosin structure organization; Brown – cellular component morphogenesis. [file 12014_2022_9364_MOESM9_ESM.pptx]

## Slide 1
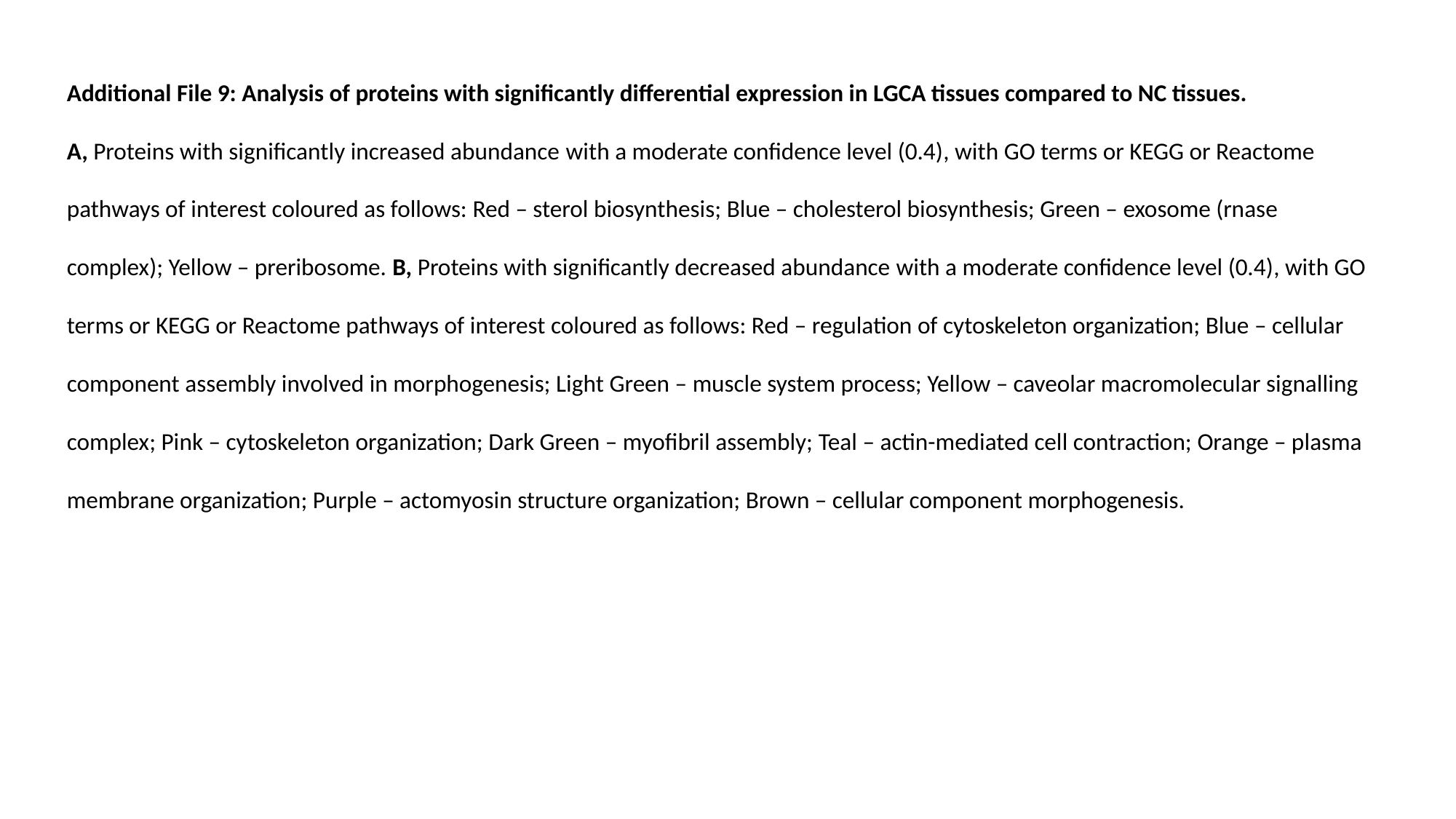

Additional File 9: Analysis of proteins with significantly differential expression in LGCA tissues compared to NC tissues.
A, Proteins with significantly increased abundance with a moderate confidence level (0.4), with GO terms or KEGG or Reactome pathways of interest coloured as follows: Red – sterol biosynthesis; Blue – cholesterol biosynthesis; Green – exosome (rnase complex); Yellow – preribosome. B, Proteins with significantly decreased abundance with a moderate confidence level (0.4), with GO terms or KEGG or Reactome pathways of interest coloured as follows: Red – regulation of cytoskeleton organization; Blue – cellular component assembly involved in morphogenesis; Light Green – muscle system process; Yellow – caveolar macromolecular signalling complex; Pink – cytoskeleton organization; Dark Green – myofibril assembly; Teal – actin-mediated cell contraction; Orange – plasma membrane organization; Purple – actomyosin structure organization; Brown – cellular component morphogenesis.

## Slide 2
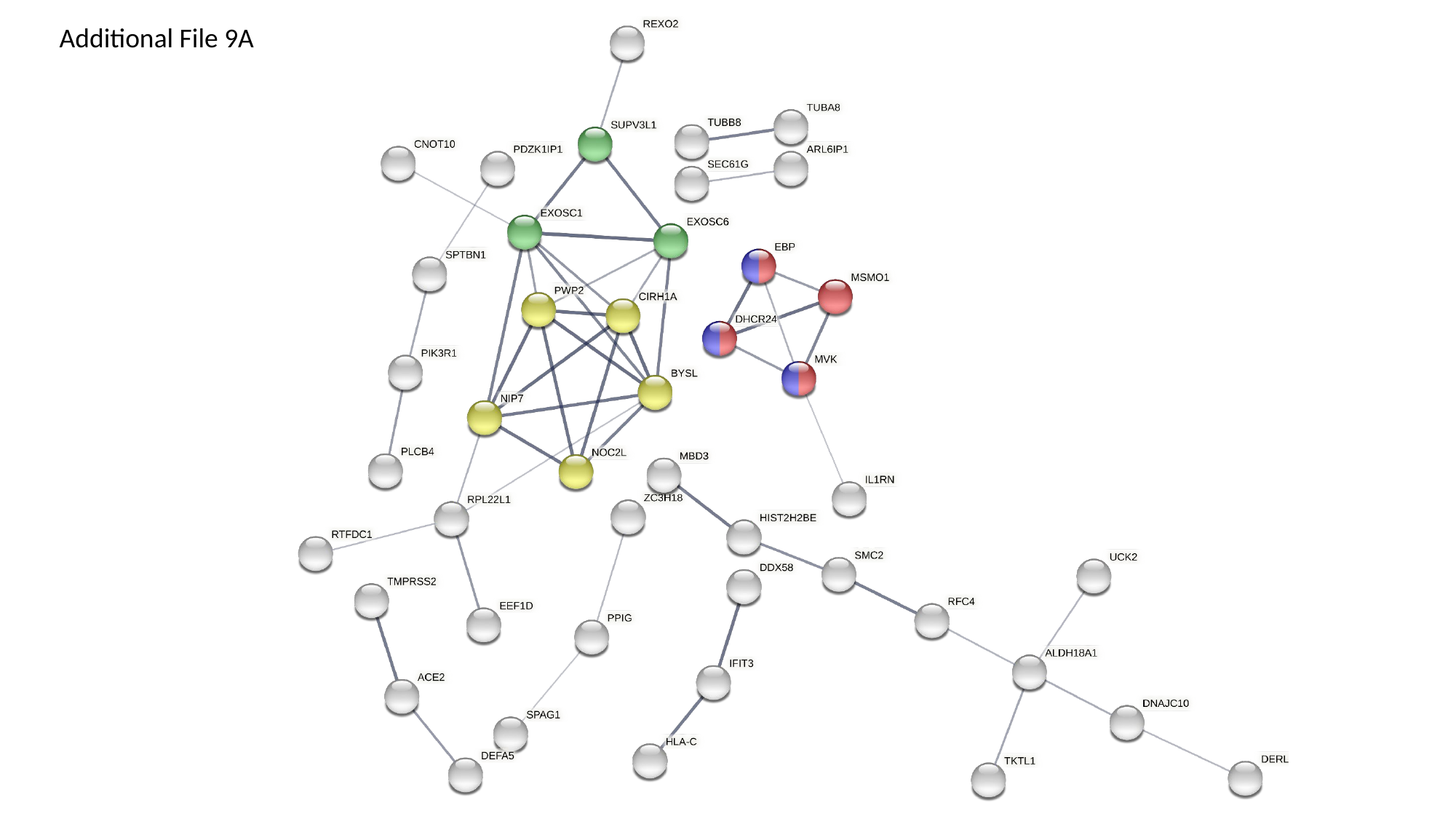

Additional File 9A

## Slide 3
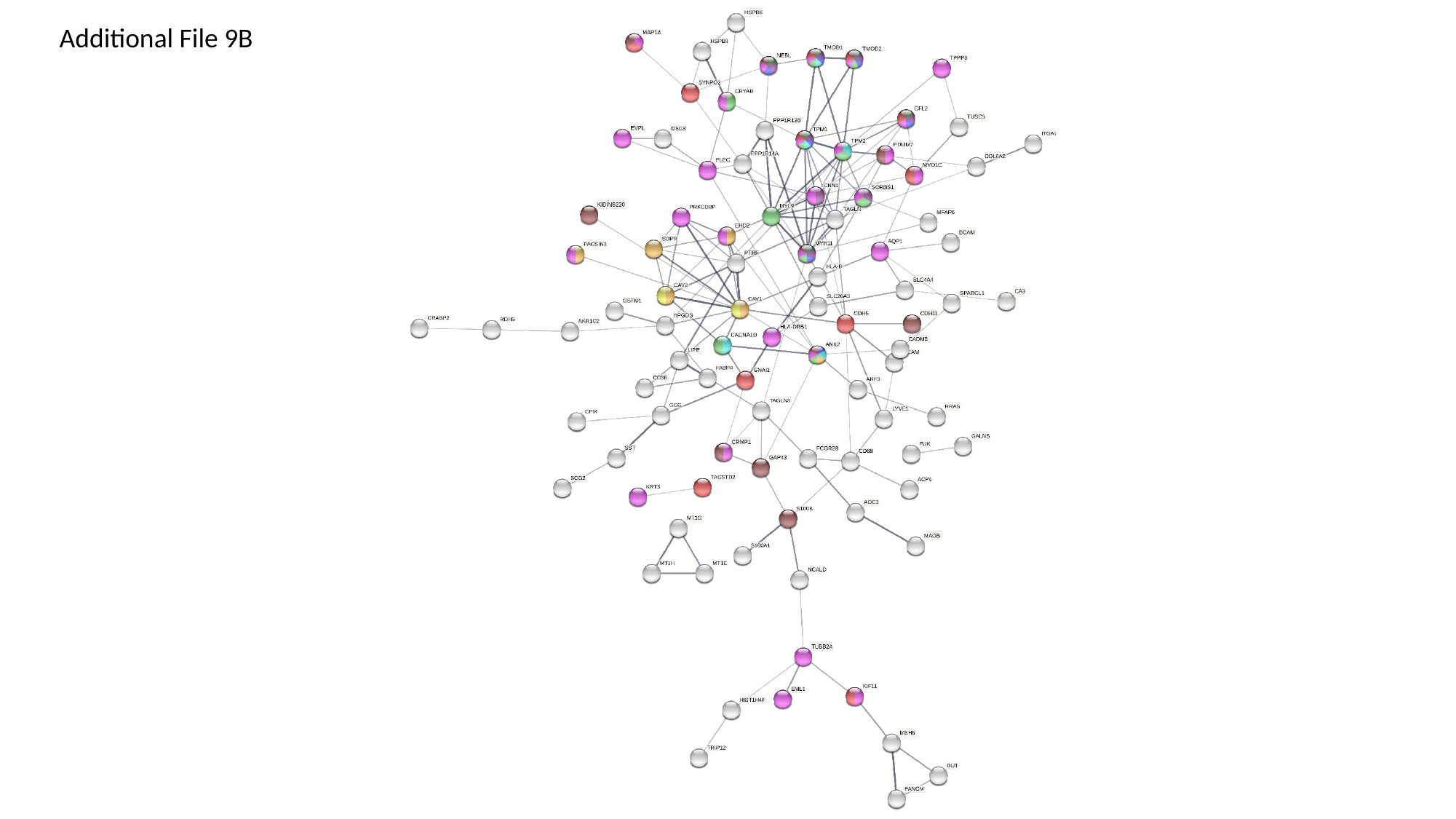

Additional File 9B
