# Supplementary material for: Proteomic analysis of low- and high-grade human colon adenocarcinoma tissues and tissue-derived primary cell lines reveals unique biological functions of tumours and new protein biomarker candidates
Source: Clin Proteomics. 2022 Jul 16;19:27. doi: 10.1186/s12014-022-09364-y (PMC9287856; doi:10.1186/s12014-022-09364-y)
Supplement: Supplementary file 10 — Additional file 10. Analysis of proteins with significantly differential expression in HGCA tissues compared to LGCA tissues. A, Proteins with significantly increased abundance with a medium confidence level (0.4), with GO terms or KEGG or Reactome pathways of interest coloured as follows: Red – detoxification; Blue – cellular zinc ion homeostasis; Light Green – regulation of growth; Yellow – immune response; Pink – immune system process; Teal – neutrophil degranulation; Orange – positive regulation of cell growth; Purple – leukocyte mediated immunity; Brown – myeloid leukocyte activation. B, Proteins with significantly decreased abundance with a medium confidence level (0.4), with GO terms or KEGG or Reactome pathways of interest coloured as follows: Red – nitrogen metabolism; Blue – tyrosine metabolism; Yellow – drug metabolism cytochrome P450. [file 12014_2022_9364_MOESM10_ESM.pptx]

## Slide 1
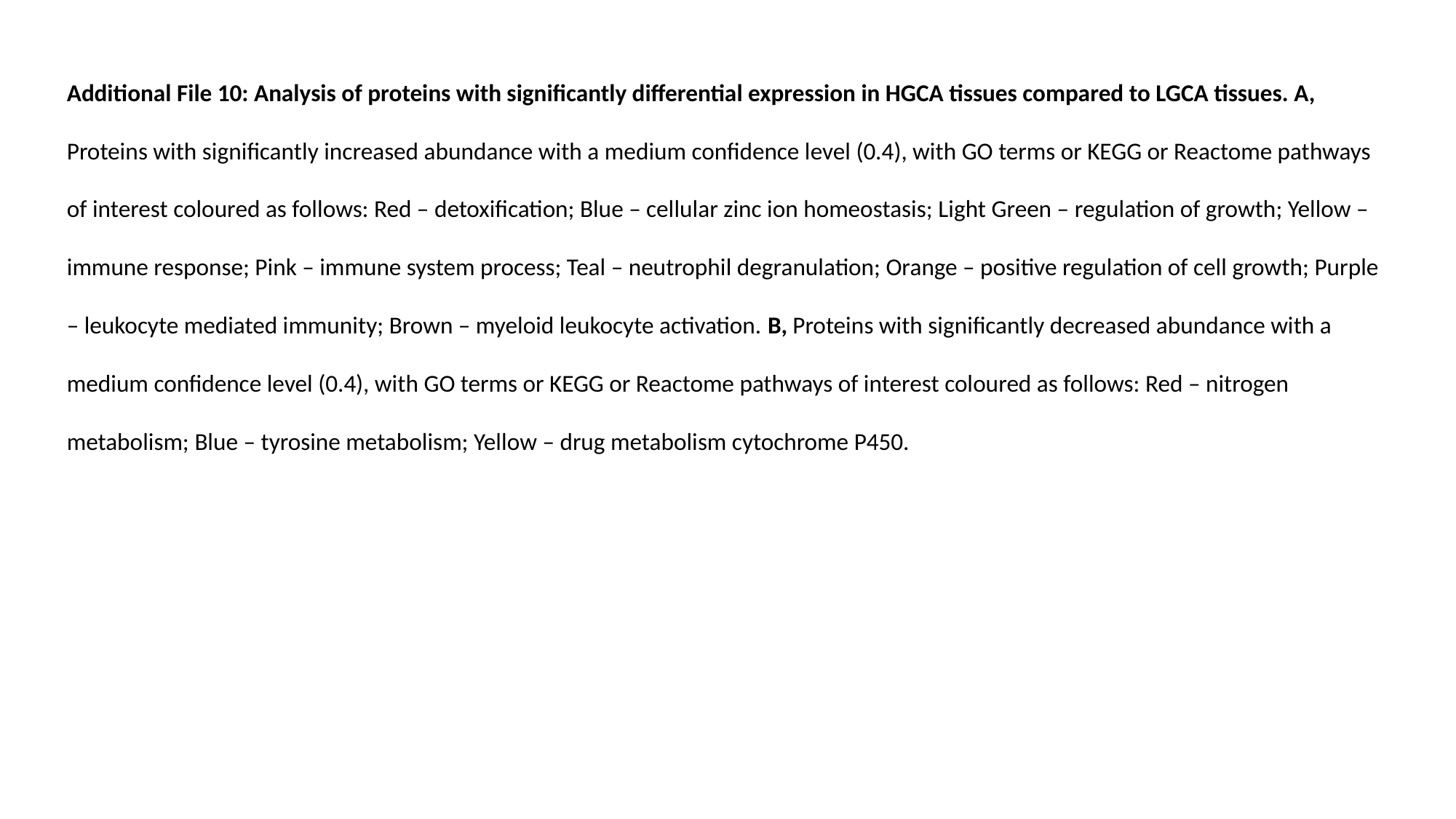

Additional File 10: Analysis of proteins with significantly differential expression in HGCA tissues compared to LGCA tissues. A, Proteins with significantly increased abundance with a medium confidence level (0.4), with GO terms or KEGG or Reactome pathways of interest coloured as follows: Red – detoxification; Blue – cellular zinc ion homeostasis; Light Green – regulation of growth; Yellow – immune response; Pink – immune system process; Teal – neutrophil degranulation; Orange – positive regulation of cell growth; Purple – leukocyte mediated immunity; Brown – myeloid leukocyte activation. B, Proteins with significantly decreased abundance with a medium confidence level (0.4), with GO terms or KEGG or Reactome pathways of interest coloured as follows: Red – nitrogen metabolism; Blue – tyrosine metabolism; Yellow – drug metabolism cytochrome P450.

## Slide 2
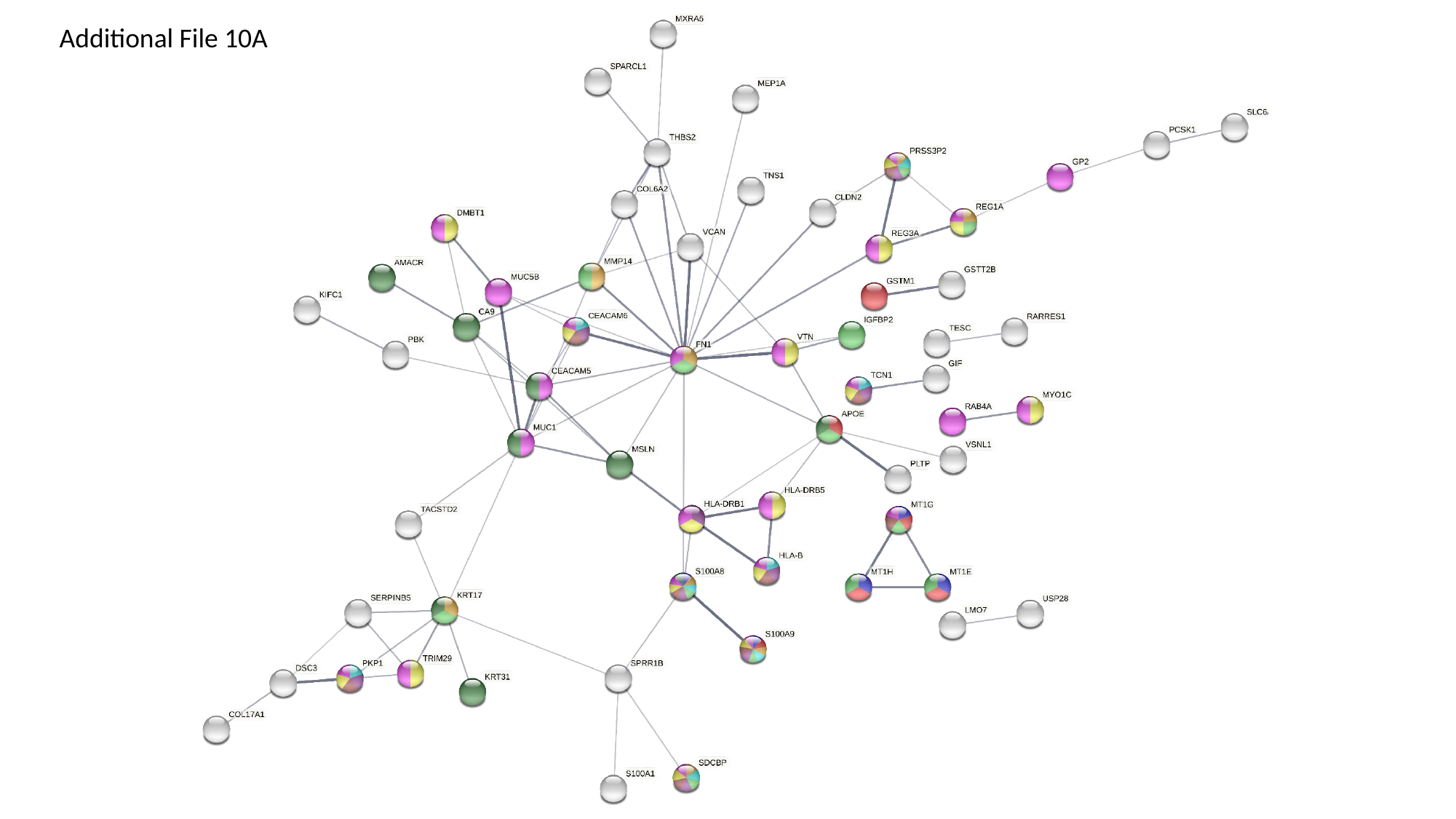

Additional File 10A

## Slide 3
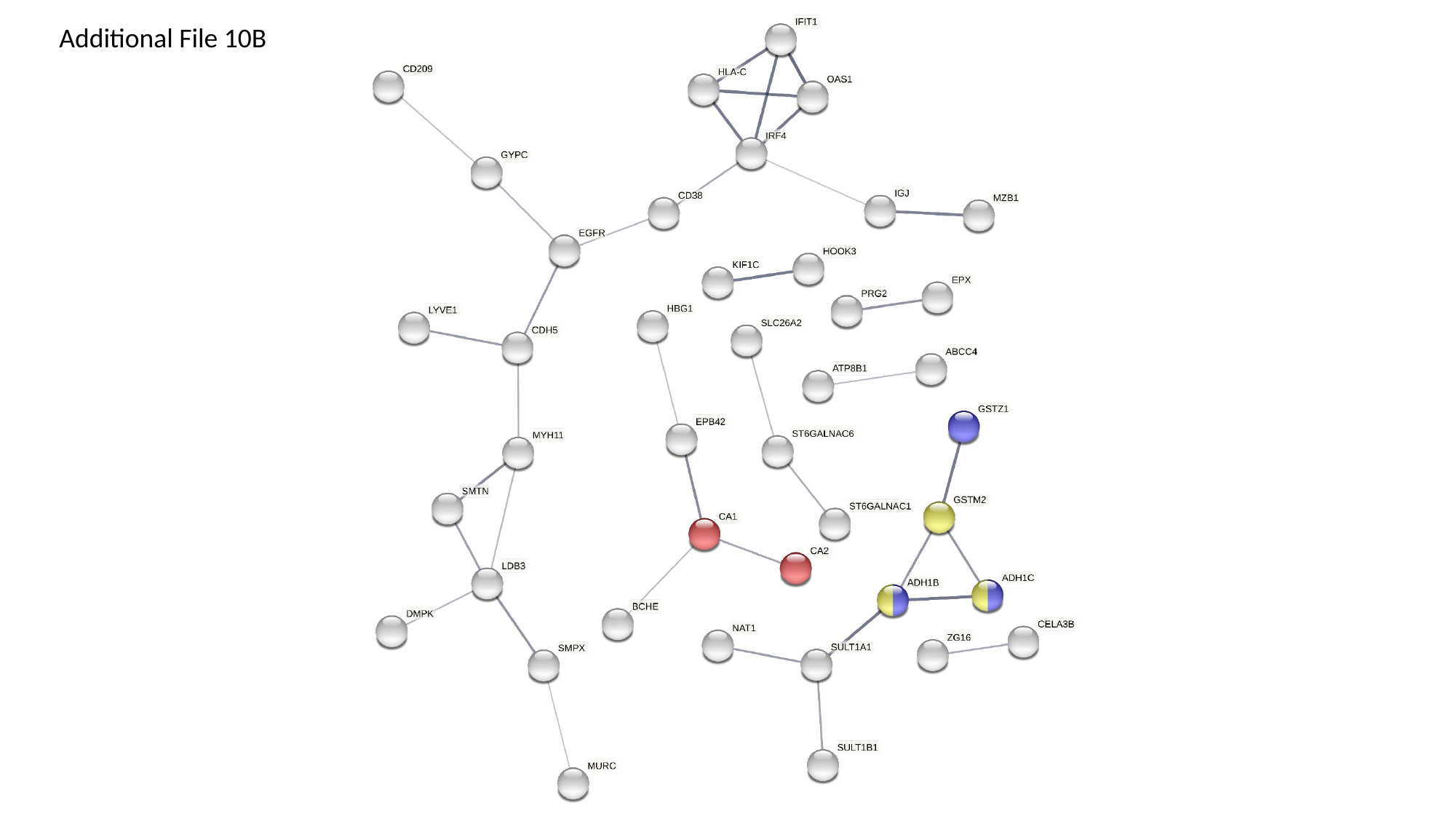

Additional File 10B
