# Supplementary material for: Proteomic analysis of low- and high-grade human colon adenocarcinoma tissues and tissue-derived primary cell lines reveals unique biological functions of tumours and new protein biomarker candidates
Source: Clin Proteomics. 2022 Jul 16;19:27. doi: 10.1186/s12014-022-09364-y (PMC9287856; doi:10.1186/s12014-022-09364-y)
Supplement: Supplementary file 11 — Additional file 11. Analysis of proteins with significantly differential expression in HGCA cell lines compared to LGCA cell lines. A, Proteins with significantly increased abundance with a medium confidence level (0.4), with GO terms or KEGG or Reactome pathways of interest coloured as follows: Red – IFN-g-mediated signalling pathway; Blue – ferroptosis; Yellow – growth plate cartilage morphogenesis; Light Green – negative regulation of leukocyte mediated immunity; Pink – response to IFN-g; Teal – regulation of i-kappab kinase/nf-kappab signaling; Orange – cellular response to chemical stress; Dark Green – cytokine-mediated signaling pathway. B, Proteins with significantly decreased abundance with a medium confidence level (0.4), with GO terms or KEGG or Reactome pathways of interest coloured as follows: Red – MHC class I protein complex; Blue – filopodium membrane; Green – dystrophin-associated glycoprotein complex; Yellow – cell adhesion; Pink – cell junction; Orange – plasma membrane protein complex. [file 12014_2022_9364_MOESM11_ESM.pptx]

## Slide 1
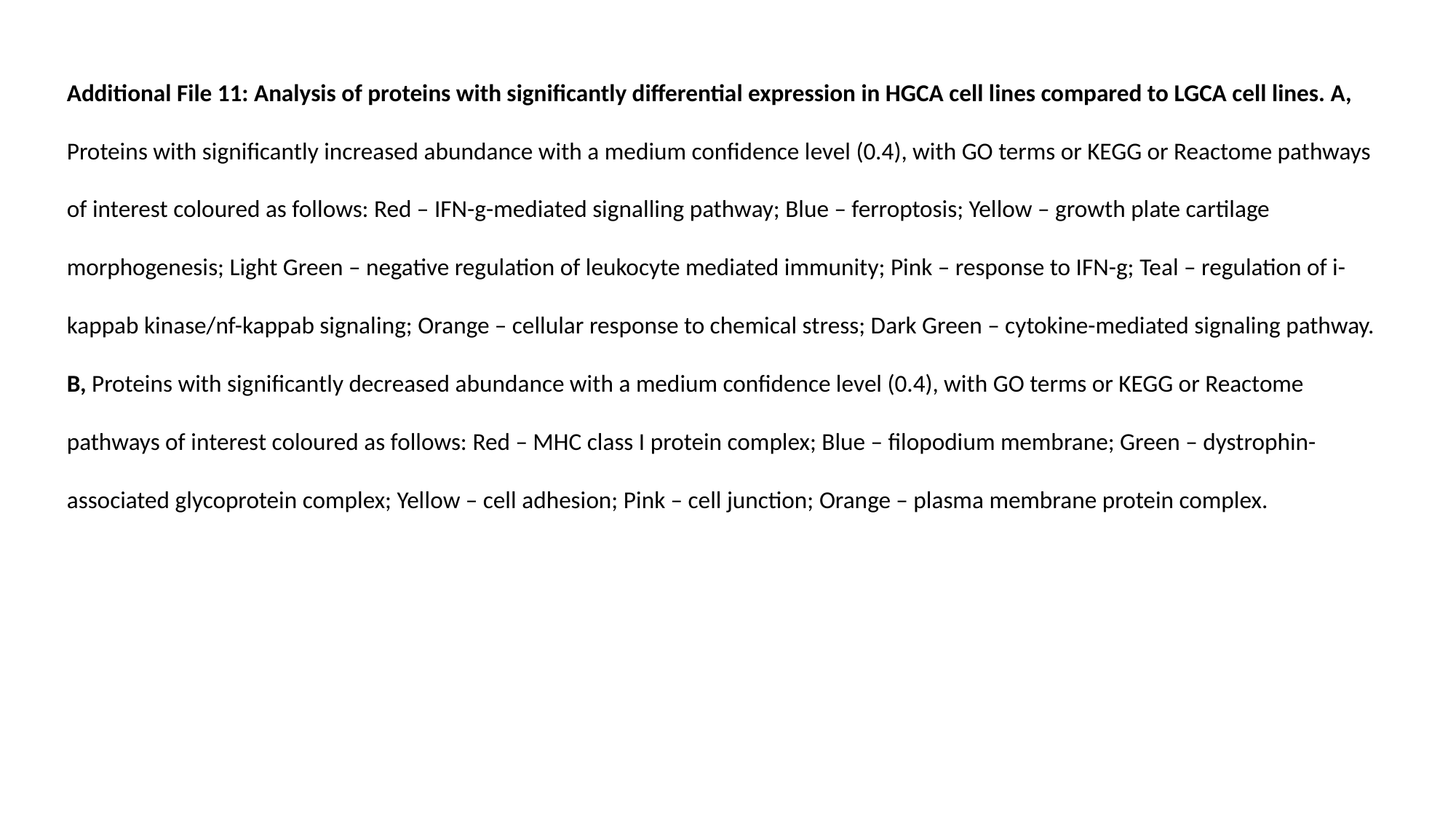

Additional File 11: Analysis of proteins with significantly differential expression in HGCA cell lines compared to LGCA cell lines. A, Proteins with significantly increased abundance with a medium confidence level (0.4), with GO terms or KEGG or Reactome pathways of interest coloured as follows: Red – IFN-g-mediated signalling pathway; Blue – ferroptosis; Yellow – growth plate cartilage morphogenesis; Light Green – negative regulation of leukocyte mediated immunity; Pink – response to IFN-g; Teal – regulation of i-kappab kinase/nf-kappab signaling; Orange – cellular response to chemical stress; Dark Green – cytokine-mediated signaling pathway. B, Proteins with significantly decreased abundance with a medium confidence level (0.4), with GO terms or KEGG or Reactome pathways of interest coloured as follows: Red – MHC class I protein complex; Blue – filopodium membrane; Green – dystrophin-associated glycoprotein complex; Yellow – cell adhesion; Pink – cell junction; Orange – plasma membrane protein complex.

## Slide 2
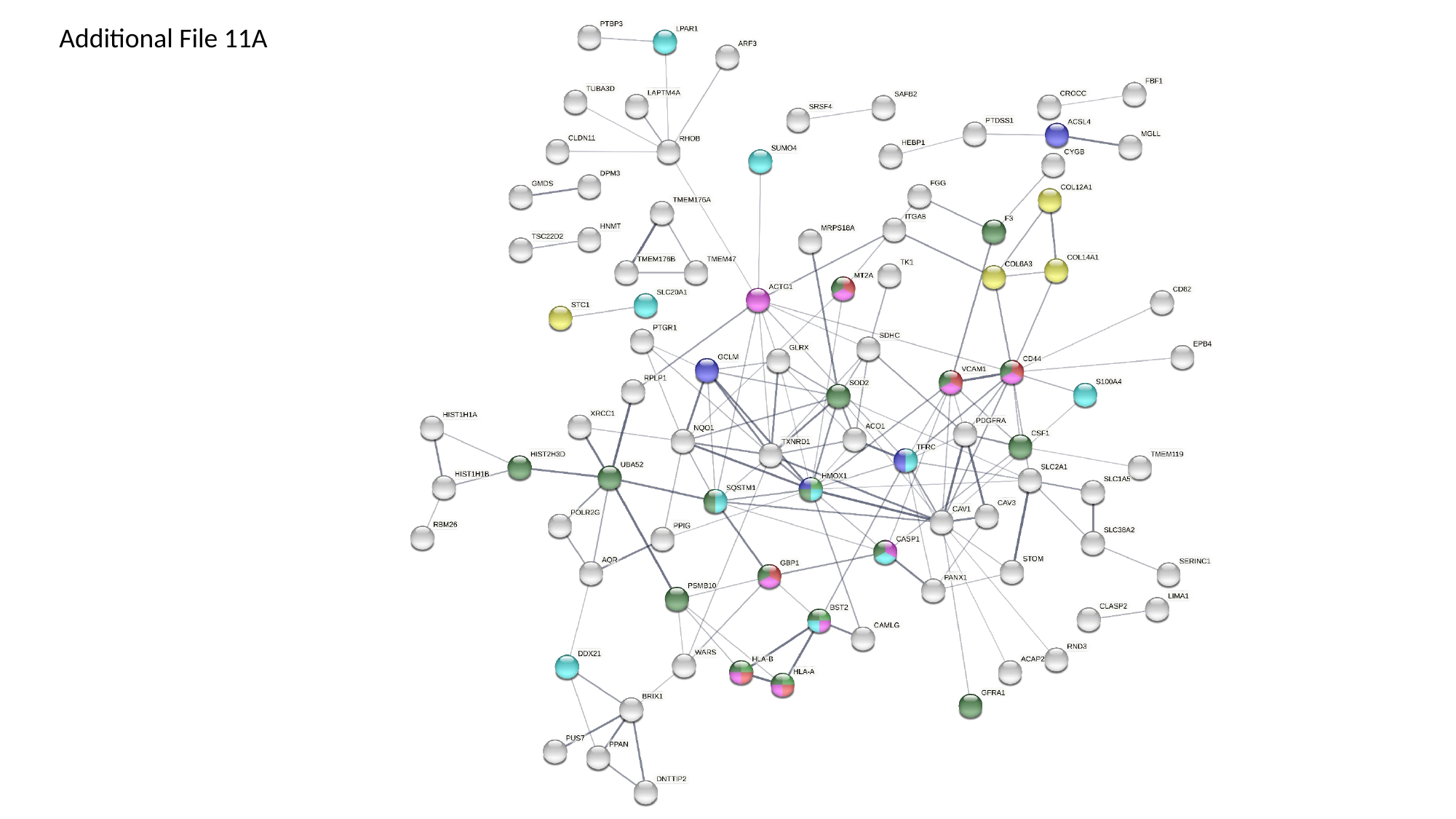

Additional File 11A

## Slide 3
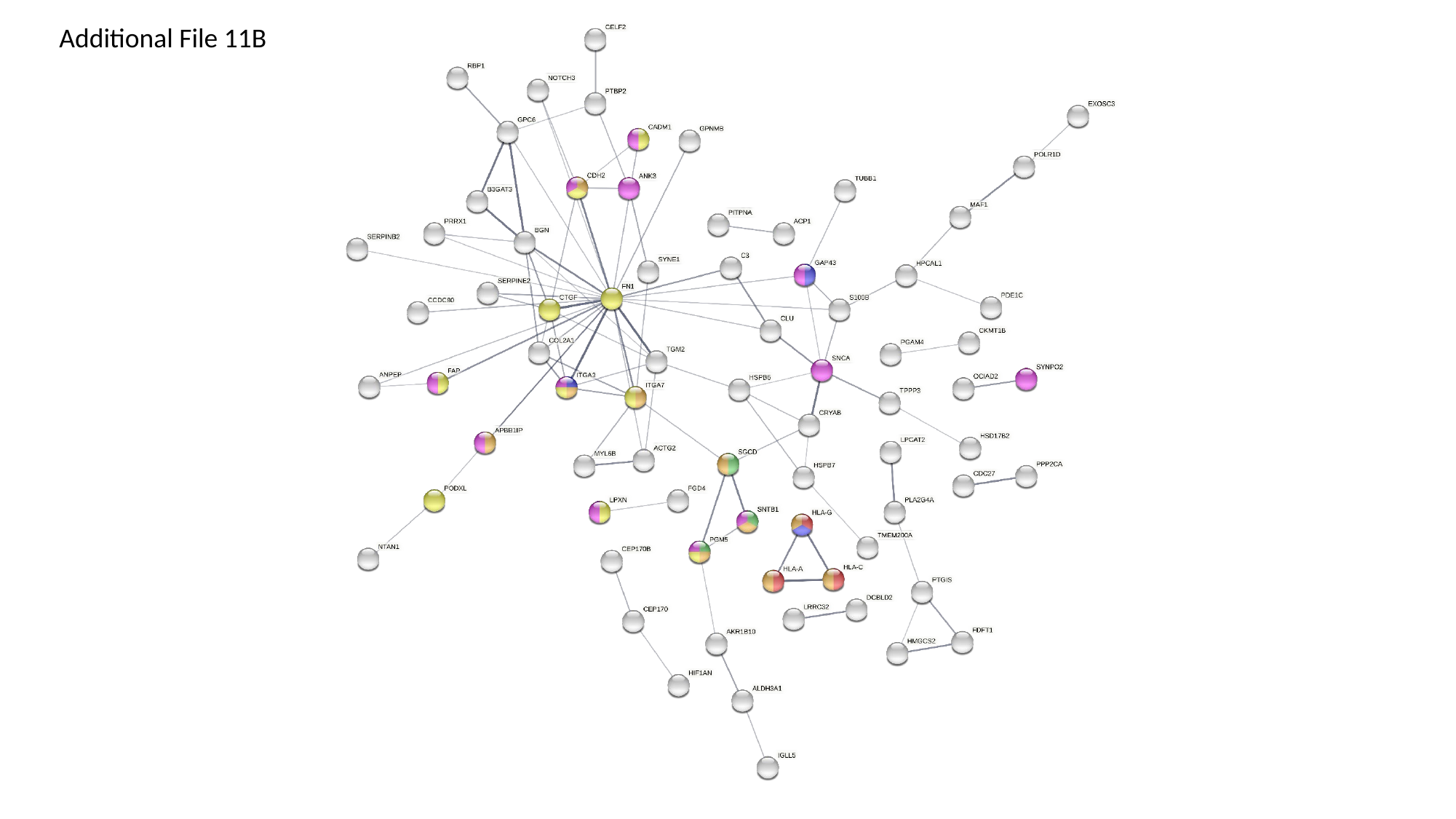

Additional File 11B
